# The plasma proteome differentiates the multisystem inflammatory syndrome in children (MIS-C) from children with SARS-CoV-2 negative sepsis

**DOI:** 10.1186/s10020-024-00806-x

**Published:** 2024-04-17

**Authors:** Maitray A. Patel, Douglas D. Fraser, Mark Daley, Gediminas Cepinskas, Noemi Veraldi, Serge Grazioli

**Affiliations:** 1 Epidemiology and Biostatistics, Western University, N6A 3K7 London, ON Canada; 2 Lawson Health Research Institute, N6C 2R5 London, ON Canada; 3 Children’s Health Research Institute, N6C 4V3 London, ON Canada; 4 Pediatrics, Western University, N6A 3K7 London, ON Canada; 5 Clinical Neurological Sciences, Western University, N6A 3K7 London, ON Canada; 6 Physiology & Pharmacology, Western University, N6A 3K7 London, ON Canada; 7 Computer Science, Western University, N6A 3K7 London, ON Canada; 8 Medical Biophysics, Western University, N6A 3K7 London, ON Canada; 9 Department of Pediatrics, Gynaecology and Obstetrics, Faculty of Medicine, University of Geneva, Geneva, Switzerland; 10 Division of Neonatal and Pediatric Intensive Care, Department of Child, Woman, and Adolescent Medicine, Geneva University Hospitals, Geneva, Switzerland; 11 London Health Sciences Centre, Room C2-C82, 800 Commissioners Road East, N6A 5W9 London, ON Canada

**Keywords:** MIS-C, Sepsis, Targeted proteomics, Machine learning, Biomarker discovery

## Abstract

**Background:**

The Multi-System Inflammatory Syndrome in Children (MIS-C) can develop several weeks after SARS-CoV-2 infection and requires a distinct treatment protocol. Distinguishing MIS-C from SARS-CoV-2 negative sepsis (SCNS) patients is important to quickly institute the correct therapies. We performed targeted proteomics and machine learning analysis to identify novel plasma proteins of MIS-C for early disease recognition.

**Methods:**

A case-control study comparing the expression of 2,870 unique blood proteins in MIS-C versus SCNS patients, measured using proximity extension assays. The 2,870 proteins were reduced in number with either feature selection alone or with a prior COMBAT-Seq batch effect adjustment. The leading proteins were correlated with demographic and clinical variables. Organ system and cell type expression patterns were analyzed with Natural Language Processing (NLP).

**Results:**

The cohorts were well-balanced for age and sex. Of the 2,870 unique blood proteins, 58 proteins were identified with feature selection (FDR-adjusted *P* < 0.005, *P* < 0.0001; accuracy = 0.96, AUC = 1.00, F1 = 0.95), and 15 proteins were identified with a COMBAT-Seq batch effect adjusted feature selection (FDR-adjusted *P* < 0.05, *P* < 0.0001; accuracy = 0.92, AUC = 1.00, F1 = 0.89). All of the latter 15 proteins were present in the former 58-protein model. Several proteins were correlated with illness severity scores, length of stay, and interventions (LTA4H, PTN, PPBP, and EGF; *P* < 0.001). NLP analysis highlighted the multi-system nature of MIS-C, with the 58-protein set expressed in all organ systems; the highest levels of expression were found in the digestive system. The cell types most involved included leukocytes not yet determined, lymphocytes, macrophages, and platelets.

**Conclusions:**

The plasma proteome of MIS-C patients was distinct from that of SCNS. The key proteins demonstrated expression in all organ systems and most cell types. The unique proteomic signature identified in MIS-C patients could aid future diagnostic and therapeutic advancements, as well as predict hospital length of stays, interventions, and mortality risks.

**Supplementary Information:**

The online version contains supplementary material available at 10.1186/s10020-024-00806-x.

## Introduction

Coronavirus disease (COVID-19) is a result of a severe acute respiratory syndrome coronavirus 2 (SARS-CoV-2) infection [1, 2]. Most children infected with SARS-CoV-2 present with mild COVID-19 symptoms, including headache, fever, and cough [3–6]; however, a subset will develop a multisystem inflammatory syndrome (MIS-C) with a prevalence of approximately 3 per 10,000 children [7–15]. MIS-C typically occurs 2–6 weeks post-infection, resulting in hyperinflammation, organ dysfunction, rashes, pain, and a high fever [9–11, 16]. A large proportion of MIS-C patients present with cardiac (myocarditis and pericarditis) and/or gastrointestinal involvement [12, 17, 18]. The pathophysiology of MIS-C is still unknown, but it shares similarities with Kawasaki Disease or Toxic Shock Syndrome [8, 11, 19–22].

The diagnosis of MIS-C is subjective, as specific disease biomarkers for MIS-C are lacking. Clinicians rely on patient history, physical examination, SARS-CoV-2 positivity using polymerase chain reaction or serology, and standard hospital laboratory testing (e.g., complete blood count, C-reactive protein, ferritin, etc.). The treatment of MIS-C follows recommended guidelines (https://cps.ca/en/documents/position/pims, 2023-05-31), which are distinct from SARS-CoV-2 negative sepsis (SCNS) treatment, and include corticosteroids, immunoglobulins, and/or anticoagulation. Severely ill MIS-C patients require admission to the pediatric intensive care unit (PICU) for advanced monitoring and additional interventions, such as ventilation and inotropic or vasopressor support. Despite optimal therapy, the long-term consequences of MIS-C may include muscular fatigue, neurological sequelae, and myocardial scars [11, 12, 17, 23, 24].

Given the subjective nature of the MIS-C diagnosis and the specific MIS-C treatment guidelines, distinguishing MIS-C from SCNS early in the disease course is critical for optimal management. Thus, the primary goal of this study was to identify blood proteins specific to MIS-C inpatients relative to age- and sex-matched SCNS patients. Our specific objectives were: (1) to measure a large number of blood proteins from each cohort with targeted proteomics [25–30]; (2) to determine the relative importance of the proteins with machine learning to differentiate MIS-C subjects; (3) to correlate protein expression with clinically relevant MIS-C variables; and (4) to determine the cell types and organ systems in which the important proteins are expressed.

## Methods

### Study participants and blood sampling

MIS-C patients admitted to the PICU were prospectively enrolled in the Geneva University Hospitals (Geneva, Switzerland), and SCNS PICU patients and healthy control subjects were enrolled in the Children’s Hospital, London Health Sciences Centre (London, ON, Canada). SARS-CoV-2 status was confirmed or ruled out for all PICU patients based on standard hospital testing using a polymerase chain reaction [31]. Blood sampling for both MIS-C and SCNS patients began on PICU admission Day 1 and continued for MIS-C patients on additional PICU days. Daily blood was obtained from ICU patients via indwelling catheters, and if a venipuncture was required, research blood draws were coordinated with a clinically indicated blood draw. In keeping with accepted research phlebotomy protocols, blood draws did not exceed maximal volumes for age. Blood was centrifuged and plasma isolated, aliquoted at 250 µL, and frozen at − 80 °C. All samples remained frozen until use, and freeze-thaw cycles were avoided. The healthy control subjects were individuals without disease, acute illness, or prescription medications that were previously banked at the Translational Research Centre, London, ON (Directed by Dr. D.D. Fraser; https://translationalresearchcentre.com/) [32, 33]. These latter samples were obtained prior to the emergence of SARS-CoV-2 in our region and, therefore, were considered not to have been exposed to the virus.

### Patient demographics and clinical data

Baseline characteristics were recorded for MIS-C and SCNS patients and included age, sex, weight, clinical measures, infectious source and pathogen, interventions, and outcomes (reported as median (IQR) for continuous variables and frequency (%) for categorical variables). Illness severity scores were calculated, including the Pediatric Risk of Mortality III (PRISM III) score, the Pediatric Index of Mortality 2 (PIM 2) score, the daily Pediatric Logistic Organ Dysfunction 2 (PELOD-2) score, and the Glasgow Coma Scale (GCS).

### Proximity extension assay

Plasma underwent proximity extension assay (PEA) as previously described at an Olink-certified laboratory (Boston, MA) [34, 35]. Specifically, we measured a total of 3072 plasma proteins in the plasma of MIS-C and SCNS patients, as well as healthy control subjects. The Olink Explore 3072 library consists of multiple panels with duplicate proteins, leading to the measurement of 2870 unique proteins. The PEA was performed in three steps: (1) antibody pairs, labeled with unique DNA oligonucleotides, were attached to their target antigen in plasma; (2) oligonucleotides that were brought into proximity hybridized and were extended by a deoxyribonucleic acid (DNA) polymerase; and (3) the newly formed DNA barcode was amplified for high-sensitivity, high-specificity readout with next generation sequencing (NovaSeq Platform; Illumina Inc., San Diego, CA). The data were generated and expressed as relative quantifications on the log2 scale of normalized protein expression (NPX) values. Samples were screened based on quality controls for immunoassay and detection, as well as the degree of hemolysis. Following proteomic quality control, all patients/subjects were deemed suitable for analysis.

### Non-parametric statistical methods

Two group comparisons were made using a Mann-Whitney U test, while three group comparisons used a Kruskall-Wallis test. A Wilcoxon Signed Rank test was used for time course analyses to account for the inter-patient variation between the different PICU days. Continuous patient variables were compared to the leading proteins with a Spearman correlation.

### Feature selection

Normalized protein expression was exponentially converted, and was then subjected to feature selection with or without a COMBAT-Seq batch-effect adjustment. The latter method decreases the potential variation caused by differences in technical factors during sample collection and processing [36]. COMBAT-Seq was performed using R statistical software (v4.3.2, [37]) with the Surrogate Variable Analysis package (v.3.50.0, [38]). In both analytic approaches, proteins with a *P* < 0.0001 and with a Benjamini Hochberg False Discovery Rate (FDR) adjusted hypothesis test *P* < 0.05 between MIS-C and SCNS patients formed reduced subsets for focused analyses.

### Machine learning

The two reduced protein sets (feature selection alone or COMBAT-Seq batch effect adjusted feature selection) were analyzed with a Random Forest, which is based on decision trees, to classify the MIS-C patients in comparison to SCNS. To reduce overfitting and maintain a conservative model, three-fold cross-validation with a Random Forest of 10 trees and a maximum depth of three was used [39]. Receiver operating characteristic (ROC) curves and Area-under-the-curve (AUC) were determined as an aggregate measure of protein performance across all possible classification thresholds [40]. Precision and Recall were determined, including their combined metric (F1 score), which was calculated as the harmonic mean. A high F1 score indicated that both Precision and Recall were high.

The protein data was visualized with a nonlinear dimensionality reduction on the full, reduced, and optimal datasets using the t-distributed stochastic nearest neighbor embedding (t-SNE) algorithm. t-SNE assumes that the ‘optimal’ representation of the data lies on a manifold with complex geometry, but a low dimension embedded in the full-dimensional space of the raw data [41]. A pairwise comparison, using cosine similarity, was conducted to determine the similarity between subjects across the selected proteins [42]. As such, subjects similar across their selected protein profile have a score closer to 1, while dissimilar subjects have a score closer to 0. The similarity analysis was done with data Min-Max scaled between 0 and 1, and the cosine similarities were visualized using a heatmap.

The sensitivity and specificity of individual proteins comparing SCNS and MIS-C on Day 1 were determined via logistic regression. A bootstrap method of 1000 repetitions with resampling with replacement and three-fold cross-validation was used to determine the average ROC curve AUC and F1 score of the individual proteins. The machine learning analysis was conducted using Python v3.10.4, Scikit-Learn v1.1.1, Scikit-Posthocs = 0.7.0, and Scipy v1.7.3, [43–45].

### Natural language processing

Exploratory expression analysis was also conducted to determine physiological areas of interest in MIS-C inpatients. Protein expression tissue specificity was parsed from the UniProt Knowledgebase using the UniProt website REST API [46]. The tissue specificity was unstructured text on the expression at the mRNA or protein level in cells or tissues gathered manually by experts. The expression information was processed by Natural Language Processing (NLP) using the Stanza Python package implemented with spaCy (Python v. 3.10.4; spaCy v. 3.3.1; spaCy-Stanza v. 1.0.2; negspaCy v. 1.0.3) [47–49]. An NLP named-entity recognition pipeline was configured with the MIMIC package for preprocessing, negation detection, and the pretrained Stanza BioNLP13CG Biomedical model. The negation detection was done using the NegEx-based negspaCy implementation with a modified English clinical term set to filter negative expression terms. Although the BioNLP13CG biomedical model was based on cancer genetics and publicly available PubMed abstracts, in comparison to the other Stanza models, it provided the most granular entity classification. The model separately identifies cell types as well as anatomical systems, organs, tissues, and multi-level tissues, which were manually combined into an organ system classification. The detected organ system and cell type entities were manually classified into keyword-based groups separately. The frequency of the keyword-based categories with respect to the relevant proteins was determined to identify physiological patterns of expression.

## Results

A total of 3 age- and sex-matched cohorts were included, consisting of MIS-C patients (median years old = 12; IQR = 3; *n* = 12), SCNS patients (median years old = 12.5; IQR = 2.2; *n* = 12) and healthy control subjects (median years old = 12.5; IQR = 4.3; *n* = 12). There were no significant differences between the ages (Kruskal-Wallis H-test, *P* = 0.8561) and sex (Chi-square, *P* = 1.000) between the three participant groups. Baseline demographics, clinical characteristics, infection source and pathogen, interventions, and outcomes are provided in Table 1.

**Table 1:** Demographics and clinical data

Variable	MIS-C Patients	SCNS Patients	P Value
Age (yrs), median (IQR)	12.0 (10.0–13.0)	12.5 (11.0-13.2)	0.617
Male sex, no. (%)	11 (91.7)	11 (91.7)	1.000
Weight (kg), median (IQR)	49.0 (36.7–57.5)	35.4 (31.8–48.7)	0.326
**Clinical Measures, median (IQR)**			
PRISM III Score	5.5 (2.8–7.3)	6.5 (2.2–13.8)	0.663
PIM2 Mortality Risk	1.2 (1.0-4.4)	-3.4 (-4.3–2.8)	**< 0.001**
dPELOD Initial	1 (0.0–3.0)	10.5 (0.0–12.0)	0.063
sPELOD Score Highest	2.5 (0.8–3.2)	12.0 (11.0-12.5)	**< 0.001**
GCS Admission	15.0 (15.0–15.0)	14.0 (7.0–15.0)	**0.007**
**Source of Infection, no. (%)**			
Respiratory	12 (100.0)	8 (66.7)	0.093
Cardiovascular	0 (0)	2 (16.7)	0.478
Gastrointestinal	0 (0)	1 (8.3)	1.000
Wound	0 (0)	1 (8.3)	1.000
**Pathogen Type, no. (%)**			
Bacteria - Gram positive	0 (0)	2 (16.7)	0.478
Bacteria - Gram negative	0 (0)	6 (50.0)	**0.014**
Viral^1^/Bacteria Gram positive	0 (0)	1 (8.3)	1.000
Viral postive^1^	0 (0)	3 (25.0)	0.217
SARS-CoV-2 positive	12 (100)	0 (0)	**< 0.001**
**Ventilation, no. (%)**			
Invasive Ventilation	2 (16.7)	6 (50.0)	0.192
Non-Invasive Ventilation	2 (16.7)	2 (16.7)	1.000
**Hemodynamic Support, no. (%)**			
Inotrope/Vasopressors	8 (66.7)	10 (83.3)	0.640
**Outcomes, median (IQR)**			
Alive, no. (%)	12 (100.0)	12 (100.0)	1.000
PICU length of stay	5.5 (2.0-7.5)	5.5 (2.8–10.2)	0.706
Hospital length of stay	8.0 (5.8–10.5)	12.5 (9.5–16.8)	**0.040**

*Note* Continuous variables compared with Mann-Whitney U Test. Binary variables compared with Fisher Exact Test. ^1^SARS-CoV-2 negative viral illness

**Fig. 1:**
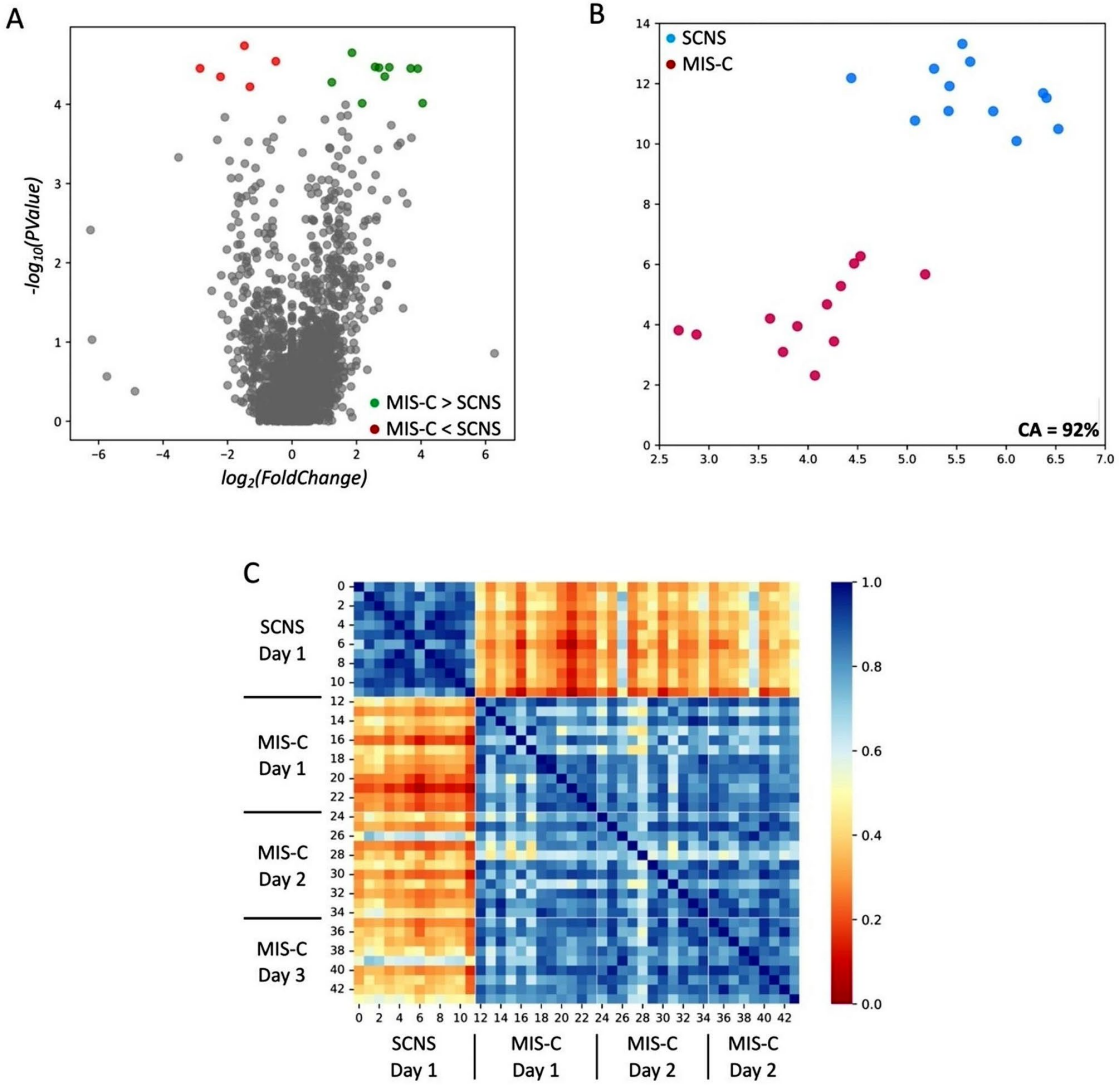
Identification of 15 key proteins differentiating MIS-C from SCNS patients. (**A**) Volcano plot demonstrating the log2 change in biomarkers between patients with MIS-C and SCNS. The top 15 biomarkers (*P* < 0.0001; FDR adjusted P value < 0.05) are highlighted, with those coloured green demonstrating an increase in protein expression in MIS-C patients compared with SCNS patients, while those in red demonstrate a decrease in MIS-C protein expression relative to SCNS patients. (**B**) Subjects plotted in two dimensions, following t-SNE dimensionality reduction of the top 15 proteins, showed cluster separation of MIS-C patients and SCNS patients on Day 1. (**C**) A heatmap demonstrating the pairwise cosine similarity between the participants’ top 15 protein profiles. A greater cosine similarity measure between subjects indicated similar protein profiles, whereas a smaller measure indicated large differences between profiles (the measure was pseudocolored on the bar scale). The protein profile of MIS-C patients was distinctively different from that of SCNS Day 1 patients, with some homogeneity across the different days.

A total of 2,888 proteins were measured from plasma samples, with 2,870 unique proteins (duplicates removed). The data set underwent feature selection, with or without a COMBAT-Seq batch effect adjustment. The two analytic approaches identified similar protein models with excellent concordance; 58 proteins were determined with feature selection (FDR-adjusted *P* < 0.005, *P* < 0.0001; Supplemental Tables 1, 2; Supplemental Figs. 1, 2), whereas 15 proteins were identified with a COMBAT-Seq batch effect adjustment (FDR-adjusted *P* < 0.05, *P* < 0.0001; Table 2; Fig. 1A). All 15 proteins were present in the 58-protein model; a comprehensive list of the 58 proteins with reported functions is found in Supplemental Table 3.

**Table 2:** Importance of the 15 biomarkers in distinguishing MIS-C from SCNS

Rank	Assay	MIS-C Patients	SCNS Patients	pValue	ROC AUC	F1	Feature Importance
1	MRPL58	0.7 (0.5–0.8)	1.0 (1.0–2.0)	0.00003	1.00	0.82	8.89
2	LTA4H	0.8 (0.3-1.0)	0.1 (0.0-0.1)	0.00004	1.00	0.80	8.75
3	BTLA	0.4 (0.2–0.6)	1.0 (1.0–1.0)	0.00002	1.00	0.98	8.58
4	C3	1.0 (0.6-1.0)	0.2 (0.2–0.2)	0.00003	1.00	0.85	8.50
5	PDGFA	3.0 (1.8-4.0)	0.5 (0.4–0.5)	0.00003	1.00	0.84	8.43
6	F10	2.0 (2.0–2.0)	0.6 (0.5–0.8)	0.00002	1.00	0.89	8.38
7	ANGPT1	4.0 (2.0-4.2)	0.5 (0.4–0.6)	0.00003	1.00	0.94	8.37
8	CREBZF	0.1 (0.0-0.2)	1.0 (0.8-1.0)	0.00004	1.00	0.96	8.33
9	PPBP	3.0 (2.0-4.2)	0.2 (0.1–0.3)	0.00004	1.00	0.90	8.25
10	PTN	0.2 (0.1–0.5)	1.0 (1.0-2.2)	0.00004	0.99	0.90	5.43
11	BDNF	1.0 (0.9-2.0)	0.1 (0.1–0.2)	0.00004	0.99	0.89	5.31
12	SERPINI1	1.0 (0.9-1.0)	0.4 (0.3–0.6)	0.00005	0.99	0.91	3.95
13	BMP4	0.0 (0.0–0.0)	0.1 (0.1–0.1)	0.00006	0.99	0.94	3.54
14	EGF	6.0 (2.8–11.0)	0.4 (0.2–0.5)	0.00009	0.97	0.91	3.28
15	LYSMD3	0.6 (0.5–0.8)	0.1 (0.1–0.3)	0.00009	0.97	0.88	2.02

*Note* Continuous variables compared with Mann-Whitney U Test

A binary classification of the 58 proteins in MIS-C versus SCNS plasma showed a balanced accuracy = 0.96, an AUC = 1.00, and an F1 = 0.95 (all 2,870 measured proteins had an accuracy = 0.83, an AUC = 0.95, and an F1 = 0.82). With the 15-protein model, a binary analysis comparing MIS-C to SCNS plasma showed a similar performance with a balanced accuracy = 0.92, an AUC = 1.00, and an F1 = 0.89 (all 2,870 measured proteins had an accuracy of 0.79, an AUC = 91, and an F1 = 0.63). The individual ROC AUCs, F1 scores, and importance ranking of the 15 proteins are provided in Table 2. All 15 proteins had excellent individual ROC AUCs ranging from 0.97 to 1.00 and had high F1 scores of 0.80–0.98.

Using t-SNE and pairwise comparison of all 2,870 proteins, it was shown that MIS-C patients were easily separable from SCNS patients (PICU Day 1) and healthy control subjects (Supplemental Fig. 2A, C). When reduced to 58 proteins, both tSNE and pairwise comparison showed separation of cohorts (classification accuracy for the three cohorts was 92%; Supplemental Fig. 2B, D; [classification accuracy of 96% for MIS-C versus SCNS only]). Using the 15-protein model, both tSNE and pairwise comparison demonstrated clear separation of MIS-C from SCNS patients on PICU Day 1 (classification accuracy 92%; Fig. 1B and C).

A paired time course analysis of the leading 58 proteins in MIS-C plasma showed decreased expression for three proteins, SERPINA1, PRDX6, and SOST (*P* < 0.05; Supplemental Fig. 3).

**Fig. 2:**
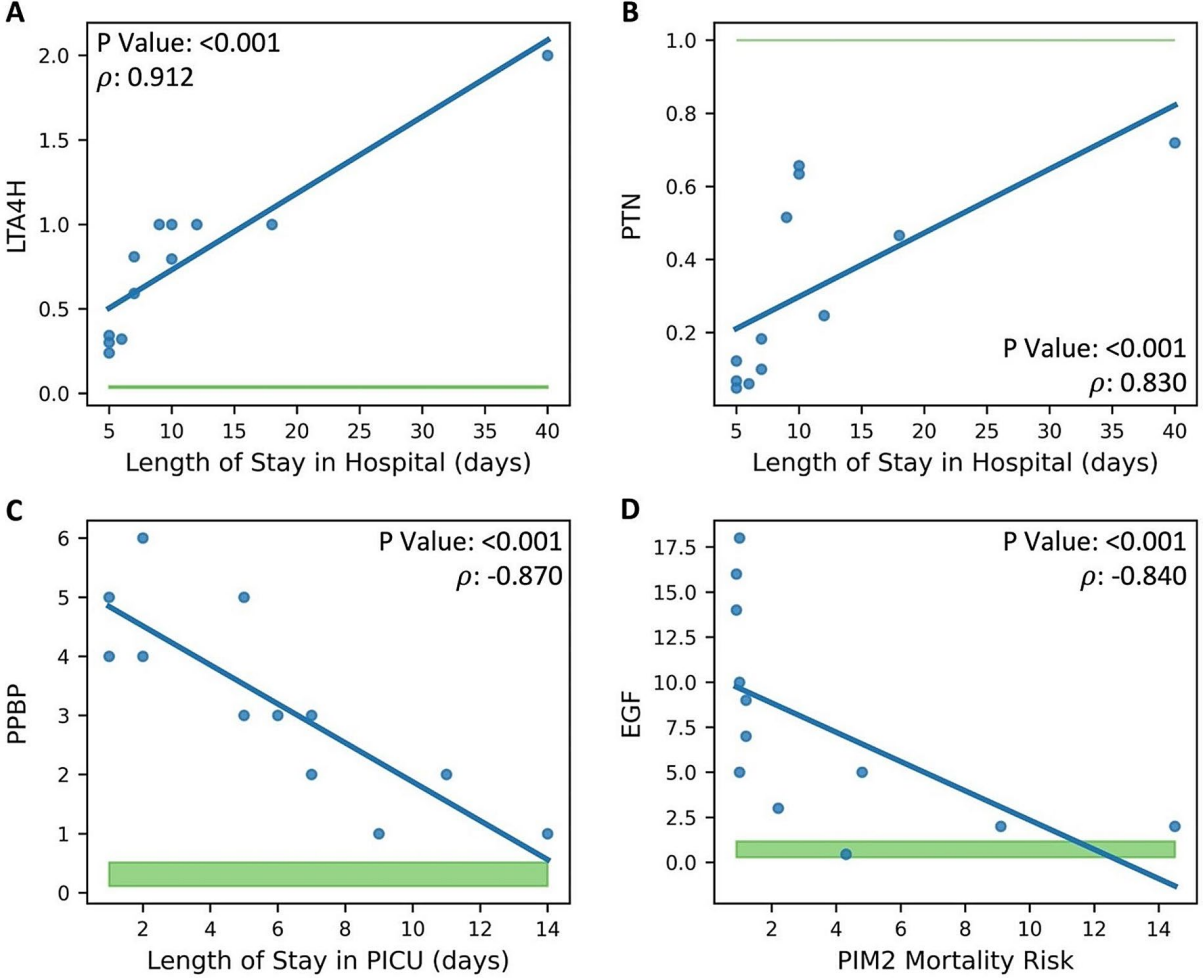
Significant Correlations Between Protein Expression in MIS-C Patients and Continuous Clinical Variables. The 15 leading proteins of MIS-C on PICU Day 1 were compared with continuous clinical and demographic variables; significant correlations are shown (*P* < 0.05). Blue points are MIS-C patient measurements; the green-filled area represents the 25th–75th percentile protein expression range of healthy control subjects. The Spearman correlation Rho statistic (𝜌) and the significance value of the comparison are shown. (**A**-**B**) Plots demonstrating a positive correlation between hospital length of stay and LTA4H and PTN such that greater expression was associated with a greater length of stay, respectively; however, compared with healthy controls, LTA4H expression was greater in all MIS-C patients on Day 1 and PTN expression was lower in all MIS-C patients on Day 1. (**C**) Plots demonstrating a negative correlation between PPBP and PICU length of stay; lower expression was associated with a greater PICU length of stay. (**D**) Plot of EGF expression demonstrating a negative correlation with PIM2 mortality risk, such that greater expression was associated with lower PIM2 mortality risk.

**Fig. 3:**
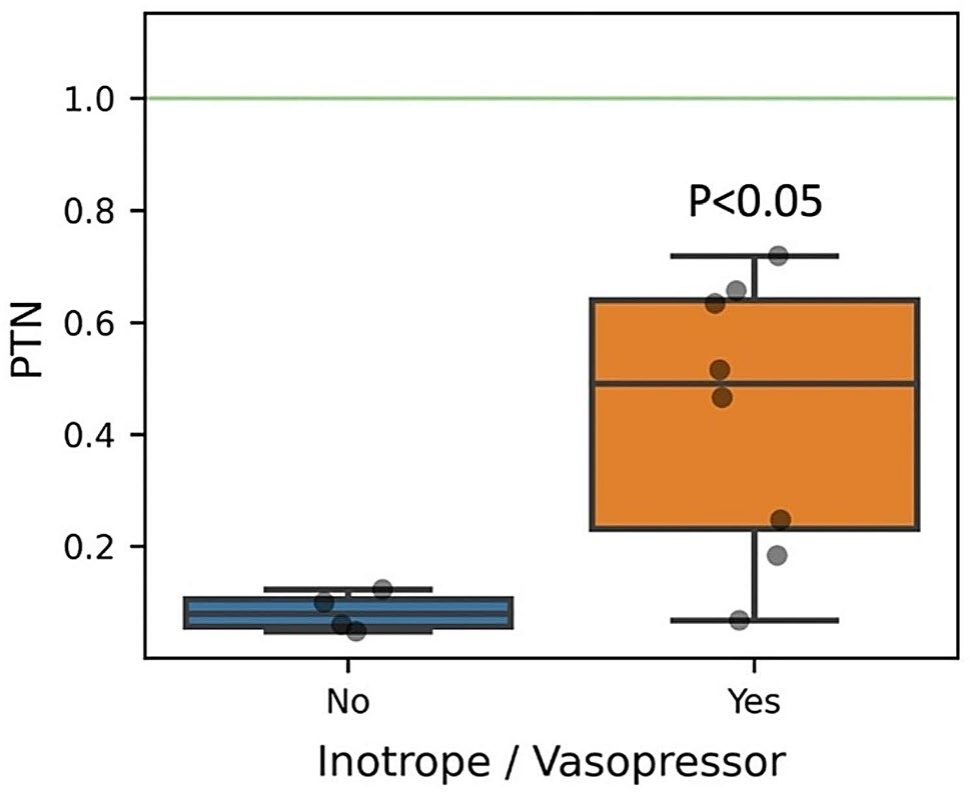
Elevated PTN Expression in MIS-C was Associated with Inotrope/Vasopressor use. The 15 leading proteins of MIS-C on PICU Day 1 were compared with the measured categorical clinical and demographic variables; significant correlations are shown (*P* < 0.05). The green-filled area represents the 25th–75th percentile protein expression range of healthy control subjects. The boxplot demonstrates elevated PTN expression in MIS-C patients that received Inotrope/Vasopressor intervention

Clinical variables of MIS-C patients were compared to the expression levels of the leading proteins; significant associations were observed for both the 58 (Supplemental Figs. 4, 5) and 15 (Figs. 2 and 3) protein models. In both datasets, the same significant correlations were found for LTA4H, PTN, PPBP, and EGF (*P* < 0.001). Hospital length of stay was positively correlated with LTA4H and PTN (Fig. 2A and B; Supplemental Fig. 4A, B), PICU length of stay was negatively correlated with PPBP (Fig. 2C; Supplemental Fig. 4D), and PIM2 mortality risk was negatively correlated with EGF expression (Fig. 2D; Supplemental Fig. 4F). MIS-C patients that received inotrope or vasopressor support had elevated PTN (*P* < 0.05; Fig. 3; Supplemental Fig. 5B). The 58-protein model also contained significant associations in MIS-C patients for ANGPT1, BDNF, FCN1, HLA-DRA, EGF and STAT5B (*P* < 0.001 to *P* < 0.05). PICU length of stay was negatively correlated with ANGPT1 (Supplemental Fig. 4C). PIM2 mortality risk score was negatively correlated with BDNF and FCN1 (Supplemental Fig. 4E, G). The sPELOD score was positively correlated with HLA-DRA (Supplemental Fig. 4H). MIS-C patients that received inotrope or vasopressor support had elevated HLA-DRA and decreased EGF (Supplemental Fig. 5A, C). Lastly, STAT5B was elevated in obese MIS-C patients (Supplemental Fig. 5D).

Using named-entity recognition on expert-curated protein expression from the UniProt knowledgebase, organ and cell-type expression was extracted and manually sorted into categories (Supplementary Tables 4, 5). Out of the 58 differentially expressed MIS-C proteins with expression information, 29 (50%) had expert-curated organ expression information, while 19 (33%) had cell-type information. The percentage of proteins expressed in an organ system and cell types is shown in Supplemental Fig. 6. The leading organ system in the MIS-C patient population was the digestive system, followed by the nervous system. Lastly, the most common cell type was leukocytes not yet determined, followed by lymphocytes, macrophages, and platelets.

## Discussion

In this study, the expression of 2,870 unique plasma proteins was measured using targeted proteomics for age- and sex-matched MIS-C patients, SCNS patients, and healthy control subjects. Feature selection identified 58 proteins that differentiated MIS-C from SCNS patients with an accuracy of 0.96, an AUC of 1.00, and a F1 of 0.95. The COMBAT-Seq batch effect adjusted feature section resulted in a 15-protein model that had similar high performance with an accuracy of 0.92, an AUC of 1.00, and an F1 of 0.89. All of the latter 15 proteins were present in the former 58-protein model. Many proteins were correlated with demographic and clinical variables, and NLP of the UniProt Knowledgebase identified protein organ and cell-type expression. Taken together, our study identified novel proteins that could be useful for early MIS-C identification to confidently administer MIS-C specific treatments, as well as predict lengths of stay, an interventions, and mortality risks. The latter may be important for resource mobilization, clinical trial stratification, and goals of care discussions with guardians.

Our MIS-C cohort was generally similar to those reported in earlier studies [12, 50–52], with only a few deviations. For example, our cohort was predominantly male (92%); however, most publications report a greater proportion of male MIS-C patients [8, 51, 53, 54]. In addition, the PRISM III and sPELOD scores were lower in our MIS-C cohort when compared to another report, suggesting less illness burden [53]. With regards to SCNS patients, they were similar to other pediatric non-SARS-CoV-2 sepsis studies in demographics, illness severity, and clinical interventions [55, 56].

The primary finding of our study was that the plasma proteome of MIS-C patients was different from that of SCNS patients. Based on a reduced number of 58 proteins, a proteomic signature for MIS-C was developed with standard feature selection, and potential disease proteins were identified. Similarly, a COMBAT-Seq batch effect adjusted feature selection demonstrated a 15-protein signature for MIS-C. In both reduced protein models, MIS-C patients were visually separable from SCNS patients and healthy control subjects with both tSNE and pairwise comparisons. An inspection of the 58 proteins that differentiate MIS-C from SCNS suggested roles in inflammation, cell growth and survival, metabolism, angiogenesis, and organ/cell-specific functions. Interestingly, over the first 3 days of PICU admission, the pairwise comparison revealed that most proteins did not change, suggesting a static disease process. The timescale analysis identified only three proteins that significantly decreased over time, including PRDX6 (a member of the peroxiredoxin family of antioxidant enzymes), SERPINA1 (a neutrophil elastase protease inhibitor), and SOST (a bone morphogenetic protein and WNT signaling inhibitor).

Correlation analyses indicate that multiple proteins were associated with MIS-C clinical variables. Four proteins (LTA4H, PTN, PPBP, and EGF) exhibited the same associations in both the standard feature selection and the COMBAT-Seq adjustment analysis. The functions of these four proteins are diverse and include angiogenesis [PTN [57]], cell growth and differentiation [PPBP, also known as CXCL7 [58], and EGF [59]], and neutrophil chemoattraction [LTA4H [60]]. Four proteins correlated with either PICU or hospital length of stays (LTA4H, PTN, ANGPT1, and PPBP), and another four proteins correlated with outcome risk scores (BDNF, EGF, FCN1, and HLA-DRA). BDNF is important for neuronal survival and plasticity [61], and FCN1 regulates innate immunity [62]. HLA-DRA mediates antigen processing by macrophages and helper T cells [63]. HLA-DRA, PTN, and EGF also differentiated those MIS-C patients that required inotrope/vasopressor support. STAT5B, a promotor of adipogenesis, differentiated MIS-C patients with pre-existing obesity [64].

Expertly curated protein expression information was collected from the UniProt Knowledgebase for the reduced 58 proteins and processed using NLP to identify organ and cell-type expression patterns. Of the 58 proteins, 29 had organ, tissue, major tissue system, and/or anatomical system information (combined to represent the organ system), and 19 had cell-type expression information. The reduced proteins were expressed in all organ systems, with the majority expressed in the digestive system. Consistent with the inflammatory and immune basis of MIS-C, the reduced proteins were primarily expressed in immune and inflammatory cells.

Gastrointestinal symptoms in MIS-C patients are common, estimated to occur in greater than 80% of all patients, with common symptoms including nausea, vomiting, diarrhea, and abdominal pain [10, 50, 65]. Our study demonstrated that the digestive system had the highest number of top proteins, as determined by NLP. Factor X (F10) was elevated in MIS-C patients, which is synthesized in the liver and is critical in the coagulation cascade to form blood clots [66]. In contrast, CREBZF, a protein that is expressed in the liver, kidney, and pancreas, was decreased in MIS-C patients. CREBZF has been associated with innate immune responses, obesity, and energy metabolism, as well as inhibiting liver regeneration [67, 68].

Neurological symptoms during acute MIS-C are prevalent, and concerns have been raised for potential long-term neurological morbidity [24, 69]. Our NLP results indicated that the nervous system is highly affected in MIS-C patients. BDNF is significantly elevated in MIS-C patients, and it is a critical synaptic protein associated with neuronal survival, plasticity, and signaling, as well as memory, learning, depression, and anxiety [70–72]. MIS-C patients with lower BDNF expression had a greater mortality risk on admission, suggesting a protective role. DCTN1, a multi-subunit protein complex that binds and activates dynein and engages retrograde axonal transport [73], was highly upregulated in MIS-C patients. Disrupted axonal transport is associated with numerous neurodegenerative diseases, including Alzheimer’s disease, Parkinson’s disease, and Huntington’s disease [74]. Both BDNF and DCTN1 have been implicated in the neurological pathology associated with COVID-19 [75, 76].

SARS-CoV-2 is a respiratory virus, and high levels of protein expression were found in the respiratory system. FCN1 was significantly elevated in the MIS-C cohort, specifically in those patients that escaped ventilation. Actively involved in innate immunity, FCN1 may protect the respiratory system through its high expression in alveolar macrophages [77, 78]. HLA-DRA was also elevated in MIS-C patients, particularly in those undergoing ventilation. As a key protein in the immune system, HLA-DRA is expressed by antigen-presenting cells, and it has been linked to bronchial epithelial cells in COVID-19 [79–81]. CA4 was elevated in the MIS-C cohort and is primarily expressed on the luminal surfaces of pulmonary capillaries, where it is known to play a critical role in gas exchange [82, 83]. In contrast to the aforementioned upregulated proteins, KLK13 expression was decreased in the MIS-C cohort and is recognized as a key protease in coronavirus HKU1 infection of pulmonary epithelial cells [84].

A high number of MIS-C-associated proteins were expressed in the musculoskeletal system, and musculoskeletal symptoms are a key characteristic of MIS-C. In fact, muscle fatigue is a possible long-term consequence of the disease process [24]. DKK1, a WNT antagonist that is a central regulator of osteoblast activity [85–87], was upregulated in our MIS-C cohort. Lower DKK1 expression in COVID-19 patients has also been linked to poorer outcomes, possibly indicating a protective effect in MIS-C patients [88]. SOST is a negative regulator for bone metabolism [89–92] and is also elevated in the MIS-C cohort, but its expression decreases by PICU Day 3. Increased SOST has been associated with disease severity in critically ill patients, including renal or hepatic organ failure [93, 94]. Decreased in the MIS-C cohort, NEB is a critical skeletal muscle protein and has been suggested to be a sepsis biomarker [95, 96].

Cardiovascular symptoms are prevalent in MIS-C and predispose patients to long-term cardiac consequences [97, 98]. Greater than 25% of the proteins with expression information were associated with the cardiovascular system. ANGPT1 is increased in the MIS-C cohort and is angiogenic with vascular protective effects [99]. As a primary regulator of angiogenesis, ANGPT1 is highly elevated in Long-COVID patients [27, 30, 100]. PPBP, also known as CXCL7, is elevated in the MIS-C cohort. Released in high amounts from activated platelets, high PPBP levels are associated with thrombosis risk [101, 102]. The expression of both ANGPT1 and PPBP in MIS-C patients was inversely correlated with PICU length of stay, suggesting a protective role linked to vascular transformation. Lastly, PDGFA and PDGFB are both elevated in the MIS-C cohort. As members of the platelet-derived growth factor family, PDGFA and PDGFB are associated with angiogenesis and vascular transformation [103–105].

NLP identified differentially expressed proteins in MIS-C patients that were associated with leukocytes, lymphocytes, and macrophages. MIS-C patients exhibited decreased expression of BTLA, an inhibitory receptor on T lymphocytes that limits T-cell proliferation and cytokine production [106]. In contrast, critically ill sepsis patients exhibit greater BTLA + T-cells [107]. The differential BTLA expression may reflect the immune response to the instigating pathogen, where a viral infection like SARS-CoV-2 requires a greater T-cell response. SAIE, upregulated in the MIS-C cohort, decreases B lymphocyte antigen receptor signaling and controls immunological tolerance [108, 109]. Its upregulation in MIS-C could either be protective by decreasing the enhanced immune response. IRAK4, a serine/threonine kinase that plays an important role in innate immunity, was upregulated in MIS-C patients. IRAK4 is upregulated in SARS-CoV-2 infection [110], and IRAK4-based silencing therapy has been proposed as a method to treat macrophage inflammatory and glycolytic reprogramming in COVID-19 [111]. Upregulated PRDX6 in the MIS-C cohort may regulate lung phospholipid metabolism, lipid peroxidation repair, and inflammatory signaling [112]. MPO, a neutrophil lysosomal protein that regulates the formation of reactive oxygen species [113], was increased in MIS-C. Variants in the MPO gene have been associated with greater MIS-C susceptibility [114–116]. Lastly, C3, CXCL11, and CCL5 are established immune proteins that were elevated in our MIS-C cohort and have been previously identified to be elevated in COVID-19 patients [117–119].

Our study identified a unique MIS-C proteome consisting of 58 proteins that were different from those of SCNS patients. Despite the novelty of our study, it had several limitations. First, the majority of patients enrolled were male. Although the generalizability of the data to females is limited, previous reports indicate MIS-C prevalence is higher in males. Second, plasma samples were collected at different tertiary pediatric hospitals and, for some samples, analyzed after international transport. To reduce sample variability, standard practices were employed for sample collection, storage, and transport. Despite these best practices, a separate parallel analysis with batch effect adjustment using COMBAT-Seq was performed to decrease possible technical variation between study hospitals. Also, conservative statistics were used to help identify proteins with a high degree of difference between cohorts. Thirdly, our study examined a limited number of matched subjects. To account for the smaller sample size, we used non-parametric statistics and conservative machine learning parameters to limit overfitting. Furthermore, reduced model building was done with a conservative filter selection to ensure a robust analysis. Fourth, while our analysis differentiated MIS-C patients from both SCNS patients, cross-identity with other diseases is a possibility. A combined model using multiple proteins might be necessary to limit cross-identity concerns. Lastly, the UniProt Knowledgebase did not have expression information on all the reduced proteins, limiting anatomical and functional understanding. Despite these limitations, our exploratory study differentiated the MIS-C cohort by its plasma proteome and provided valuable insights into MIS-C pathophysiology.

## Conclusion

Differentiation of MIS-C from SCNS early in the disease course is critical to implementing treatment guidelines, as well as predicting the length of stay and mortality risk. Our study suggests that an accurate MIS-C diagnosis can be obtained with protein assays (e.g., lateral flow assay, quantitative enzyme-linked immunosorbent assay, multiplex immunoassay, etc.). Protein assays may also aid in the identification of MIS-C treatment responders. Finally, our study sheds light on MIS-C pathophysiology, with identified proteins mediating inflammation, cell growth and survival, metabolism, angiogenesis, and organ/cell-specific functions. These exploratory results highlight the distinct aspects of MIS-C and may serve as a basis for future protein- and pathway-specific studies.

### Electronic supplementary material

Below is the link to the electronic supplementary material.

Supplementary Material 1

## Data Availability

The datasets generated and/or analysed during the current study are available from the corresponding author on reasonable request.

## Abbreviations

AUC: Area-Under-The-Curve
COVID-19: Coronavirus Disease 2019
DNA: deoxyribonucleic acid
GCS: Glasgow Coma Scale
MIS-C: Multisystem Inflammatory Syndrome in Children
MODS: Multiple Organ Dysfunction Score
NLP: Natural Language Processing
NPX: Normalized Protein Expression
PEA: Proximity Extension Assay
PELOD-2: Pediatric Logistic Organ Dysfunction 2
PICU: Pediatric Intensive Care Unit
PIM 2: Pediatric Index of Mortality 2
PRISM III: Pediatric Risk of Mortality III
RFE: Recursive Feature Elimination algorithm
ROC: Receiver Operating Characteristic Curves
SARS-CoV-2: Severe Acute Respiratory Syndrome Coronavirus 2
SCNS: SARS-CoV-2 Negative Sepsis
t-SNE: T-distributed Stochastic Nearest Neighbor Embedding algorithm

## References

[1] Yuki K, Fujiogi M, Koutsogiannaki S (2020). COVID-19 pathophysiology: a review. Clin Immunol.

[2] Alimohamadi Y, Sepandi M, Taghdir M, Hosamirudsari H (2020). Determine the most common clinical symptoms in COVID-19 patients: a systematic review and meta-analysis. J Prev Med Hyg.

[3] Russell MV (2021). Systematic review of reviews of symptoms and signs of COVID-19 in children and adolescents. Arch Dis Child.

[4] Case SM, Son MB (2021). COVID-19 in Pediatrics. Rheumatic Disease Clin North Am.

[5] Lopez-Leon S (2022). Long-COVID in children and adolescents: a systematic review and meta-analyses. Sci Rep.

[6] Thallapureddy K (2022). Long-term complications of COVID-19 infection in adolescents and children. Curr Pediatr Rep.

[7] Bukulmez H. (2021) Current understanding of Multisystem Inflammatory Syndrome (MIS-C) following COVID-19 and its distinction from Kawasaki Disease. Curr Rheumatol Rep 23. 10.1007/s11926-021-01028-4 PMC8254432 34216296

[8] Consiglio CR (2020). The Immunology of Multisystem Inflammatory Syndrome in Children with COVID-19. Cell.

[9] Gottlieb M, Bridwell R, Ravera J, Long B (2021). Multisystem inflammatory syndrome in children with COVID-19. Am J Emerg Med.

[10] Hoste L, Van Paemel R, Haerynck F. (2021) Multisystem inflammatory syndrome in children related to COVID-19: a systematic review. Eur J Pediatr. 10.1007/s00431-021-03993-5 PMC7890544 33599835

[11] Rafferty MS (2021). Multisystem inflammatory syndrome in children (MIS-C) and the coronavirus pandemic: current knowledge and implications for public health. J Infect Public Health.

[12] Feldstein LR (2020). Multisystem inflammatory syndrome in U.S. children and adolescents. N Engl J Med.

[13] Dionne A, Son MBF, Randolph AG (2022). An update on multisystem inflammatory syndrome in children related to SARS-CoV-2. Pediatr Infect Dis J.

[14] Lopez L (2022). Lower risk of multi-system inflammatory syndrome in children (MIS-C) with the omicron variant. Lancet Reg Health - Western Pac.

[15] Eleftheriou I (2023). Decreasing incidence of the Multisystem Inflammatory Syndrome in Children over 3 pandemic waves. Pediatr Infect Dis J.

[16] Feldstein LR (2021). Characteristics and outcomes of US children and adolescents with Multisystem Inflammatory Syndrome in Children (MIS-C) compared with severe Acute COVID-19. JAMA.

[17] Cantarutti N (2022). Long-Term Cardiovascular Outcome in children with MIS-C linked to SARS-CoV-2 Infection—An Italian Multicenter experience. Biology.

[18] Miller J (2020). Gastrointestinal symptoms as a major presentation component of a Novel Multisystem Inflammatory Syndrome in Children that is related to Coronavirus Disease 2019: a single Center experience of 44 cases. Gastroenterology.

[19] Whittaker E (2020). Clinical characteristics of 58 children with a Pediatric Inflammatory Multisystem Syndrome temporally Associated with SARS-CoV-2. JAMA.

[20] Pouletty M (2020). Paediatric multisystem inflammatory syndrome temporally associated with SARS-CoV-2 mimicking Kawasaki disease (Kawa-COVID-19): a multicentre cohort. Ann Rheum Dis.

[21] Klavina L et al. (2023) Comparison of characteristics and outcomes of Multisystem Inflammatory Syndrome, Kawasaki Disease and toxic shock syndrome in children. Med (Kaunas) 59. 10.3390/medicina59030626 PMC10056689 36984627

[22] Rodriguez-Smith JJ (2021). Inflammatory biomarkers in COVID-19-associated multisystem inflammatory syndrome in children, Kawasaki disease, and macrophage activation syndrome: a cohort study. Lancet Rheumatol.

[23] Abrams JY (2022). Trends in treatments for Multisystem Inflammatory Syndrome in Children (MIS-C), United States, February 2020– July 2021. Clin Infect Dis.

[24] Penner J (2021). 6-month multidisciplinary follow-up and outcomes of patients with paediatric inflammatory multisystem syndrome (PIMS-TS) at a UK tertiary paediatric hospital: a retrospective cohort study. Lancet Child Adolesc Health.

[25] Fraser DD (2020). Novel outcome biomarkers identified with targeted proteomic analyses of plasma from critically ill coronavirus Disease 2019 patients. Crit Care Explor.

[26] Iosef C (2023). COVID-19 plasma proteome reveals novel temporal and cell-specific signatures for disease severity and high-precision disease management. J Cell Mol Med.

[27] Patel MA (2023). Organ and cell-specific biomarkers of Long-COVID identified with targeted proteomics and machine learning. Mol Med.

[28] Van Nynatten LR (2022). Novel plasma protein biomarkers from critically ill sepsis patients. Clin Proteom.

[29] Miller MR (2021). Putative concussion biomarkers identified in adolescent male athletes using targeted plasma proteomics. Front Neurol.

[30] Iosef C (2023). Plasma proteome of Long-COVID patients indicates HIF-mediated vasculo-proliferative disease with impact on brain and heart function. J Transl Med.

[31] *CDC 2019-Novel Coronavirus (2019-nCoV) Real-Time RT-PCR Diagnostic Panel* [Internet]. Available from: https://www.fda.gov/media/134922/download. 10.1371/journal.pone.0260487 PMC8673615 34910739

[32] Brisson AR, Matsui D, Rieder MJ, Fraser DD (2012). Translational research in pediatrics: tissue sampling and biobanking. Pediatrics.

[33] Gillio-Meina C, Cepinskas G, Cecchini EL, Fraser DD (2013). Translational research in pediatrics II: blood collection, processing, shipping, and storage. Pediatrics.

[34] Lundberg M, Eriksson A, Tran B, Assarsson E, Fredriksson S (2011). Homogeneous antibody-based proximity extension assays provide sensitive and specific detection of low-abundant proteins in human blood. Nucleic Acids Res.

[35] Assarsson E (2014). Homogenous 96-plex PEA immunoassay exhibiting high sensitivity, specificity, and excellent scalability. PLoS ONE.

[36] Zhang Y, Parmigiani G, Johnson WE. (2020) ComBat-seq: batch effect adjustment for RNA-seq count data. NAR Genomics Bioinf 2. 10.1093/nargab/lqaa078 PMC7518324 33015620

[37] Team RC. (2023) R: A Language and Environment for Statistical Computing. In. R Foundation for Statistical Computing, Vienna, Austria.

[38] Leek Jt JWEPHSFEJJAEZYSJDTLC. (2023) sva: Surrogate Variable Analysis. In.

[39] Tang C, Garreau D, von Luxburg U. (2018) When do random forests fail? In: NeurIPS pp. 2987–97.

[40] Bradley AP (1997). The use of the area under the ROC curve in the evaluation of machine learning algorithms. Pattern Recogn.

[41] Van der Maaten L, Hinton G. (2008) Visualizing data using t-SNE. J Mach Learn Res 9.

[42] Jambu M, Jambu M (1991). Chap. 10 - classification of individuals–variables data sets. Exploratory and Multivariate Data Analysis.

[43] Pedregosa F (2011). Scikit-learn: machine learning in Python. J Mach Learn Res.

[44] Terpilowski MA (2019). scikit-posthocs: pairwise multiple comparison tests in Python. J Open Source Softw.

[45] Virtanen P (2020). SciPy 1.0: fundamental algorithms for scientific computing in Python. Nat Methods.

[46] Bateman A (2021). UniProt: the universal protein knowledgebase in 2021. Nucleic Acids Res.

[47] Zhang Y, Zhang Y, Qi P, Manning CD, Langlotz CP (2021). Biomedical and clinical English model packages for the Stanza Python NLP library. J Am Med Inform Assoc.

[48] Qi P, Zhang Y, Zhang Y, Bolton J, Manning CD. (2020) Stanza: A Python natural language processing toolkit for many human languages. *arXiv preprint arXiv:2003.07082*.

[49] Honnibal M, Montani I, Van Landeghem S, Boyd A. (2020) spaCy: Industrial-strength Natural Language Processing in Python.

[50] Ramaswamy A (2021). Immune dysregulation and autoreactivity correlate with disease severity in SARS-CoV-2-associated multisystem inflammatory syndrome in children. Immunity.

[51] Hoste L, Van Paemel R, Haerynck F (2021). Multisystem inflammatory syndrome in children related to COVID-19: a systematic review. Eur J Pediatr.

[52] Morparia K (2022). Thromboelastography profiles in critically ill children with multisystem inflammatory syndrome. Pediatr Blood Cancer.

[53] Emeksiz S (2021). Therapeutic plasma exchange: a potential management strategy for critically ill MIS-C patients in the pediatric intensive care unit. Transfus Apheres Sci.

[54] Porritt RA et al. (2021) The autoimmune signature of hyperinflammatory multisystem inflammatory syndrome in children. J Clin Invest 131. 10.1172/JCI151520 PMC8516454 34437303

[55] Zurek J, Vavrina M (2015). Procalcitonin Biomarker kinetics to predict Multiorgan Dysfunction Syndrome in Children with Sepsis and systemic inflammatory response syndrome. Iran J Pediatr.

[56] Han YY, Doughty LA, Kofos D, Sasser H, Carcillo JA (2003). Procalcitonin is persistently increased among children with poor outcome from bacterial sepsis*. Pediatr Crit Care Med.

[57] Perez-Pinera P, Berenson JR, Deuel TF (2008). Pleiotrophin, a multifunctional angiogenic factor: mechanisms and pathways in normal and pathological angiogenesis. Curr Opin Hematol.

[58] Wu Q, Tu H, Li J (2022). Multifaceted roles of chemokine C-X-C motif Ligand 7 in inflammatory diseases and Cancer. Front Pharmacol.

[59] Wee P, Wang Z. (2017) Epidermal growth factor receptor cell Proliferation Signaling pathways. Cancers (Basel) 9. 10.3390/cancers9050052 PMC5447962 28513565

[60] Snelgrove RJ (2011). Leukotriene A4 hydrolase: an anti-inflammatory role for a proinflammatory enzyme. Thorax.

[61] Miranda M, Morici JF, Zanoni MB, Bekinschtein P (2019). Brain-derived neurotrophic factor: a key molecule for memory in the healthy and the pathological brain. Front Cell Neurosci.

[62] Bidula S, Sexton DW, Schelenz S. (2019) Ficolins and the Recognition of Pathogenic Microorganisms: An Overview of the Innate Immune Response and Contribution of Single Nucleotide Polymorphisms. *J Immunol Res* 2019: 3205072. 10.1155/2019/3205072 PMC6379837 30868077

[63] Lekkou A, Karakantza M, Mouzaki A, Kalfarentzos F, Gogos CA (2004). Cytokine production and monocyte HLA-DR expression as predictors of outcome for patients with community-acquired severe infections. Clin Diagn Lab Immunol.

[64] Wakao H, Wakao R, Oda A, Fujita H (2011). Constitutively active Stat5A and Stat5B promote adipogenesis. Environ Health Prev Med.

[65] Sacco K (2022). Immunopathological signatures in multisystem inflammatory syndrome in children and pediatric COVID-19. Nat Med.

[66] Roberts HR, Hoffman M, Monroe DM (2006). A cell-based model of Thrombin Generation. Semin Thromb Hemost.

[67] Hu Z (2020). CREBZF as a Key Regulator of STAT3 pathway in the control of liver regeneration in mice. Hepatology.

[68] Hu Z (2019). 265-OR: myeloid CREBZF couples inflammatory signals to systemic insulin resistance by regulating Immune response of the adipose tissue. Diabetes.

[69] Fink EL (2022). Prevalence and risk factors of neurologic manifestations in Hospitalized Children diagnosed with Acute SARS-CoV-2 or MIS-C. Pediatr Neurol.

[70] Bathina S, Das UN (2015). Brain-derived neurotrophic factor and its clinical implications. Arch Med Sci.

[71] Miranda M, Morici JF, Zanoni MB, Bekinschtein P. (2019) Brain-derived neurotrophic factor: a key molecule for memory in the healthy and the pathological brain. Front Cell Neurosci 13. 10.3389/fncel.2019.00363 PMC6692714 31440144

[72] Martinowich K, Manji H, Lu B (2007). New insights into BDNF function in depression and anxiety. Nat Neurosci.

[73] Schroer TA (2004). Dynactin. Annu Rev Cell Dev Biol.

[74] Millecamps S, Julien JP (2013). Axonal transport deficits and neurodegenerative diseases. Nat Rev Neurosci.

[75] Petrella C et al. (2023) COVID-19 Affects Serum Brain-Derived Neurotrophic Factor and Neurofilament Light Chain in Aged Men: Implications for Morbidity and Mortality. *Cells* 12. 10.3390/cells12040655 PMC9954454 36831321

[76] Prasad K et al. (2021) Insights into the SARS-CoV-2-Mediated Alteration in the Stress Granule Protein Regulatory Networks in Humans. *Pathogens* 10. 10.3390/pathogens10111459 PMC8624858 34832615

[77] Liao M (2020). Single-cell landscape of bronchoalveolar immune cells in patients with COVID-19. Nat Med.

[78] Sheerin D (2022). Immunopathogenic overlap between COVID-19 and Tuberculosis identified from transcriptomic meta-analysis and human macrophage infection. iScience.

[79] Ma D (2021). Single-cell RNA sequencing identify SDCBP in ACE2-positive bronchial epithelial cells negatively correlates with COVID-19 severity. J Cell Mol Med.

[80] Li X (2021). Single-cell analysis reveals the Immune Characteristics of Myeloid Cells and Memory T Cells in recovered COVID-19 patients with different severities. Front Immunol.

[81] Chen K-D et al. (2022) Comparable bidirectional neutrophil immune dysregulation between Kawasaki disease and severe COVID-19. Front Immunol 13. 10.3389/fimmu.2022.995886 PMC9499176 36159873

[82] Justet A, Zhao AY, Kaminski N (2022). From COVID to fibrosis: lessons from single-cell analyses of the human lung. Hum Genomics.

[83] Gillich A (2020). Capillary cell-type specialization in the alveolus. Nature.

[84] Milewska A et al. (2020) Kallikrein 13 serves as a priming protease during infection by the human coronavirus HKU1. Sci Signal 13. 10.1126/scisignal.aba9902 PMC7857416 33234691

[85] Niida A (2004). DKK1, a negative regulator of wnt signaling, is a target of the β-catenin/TCF pathway. Oncogene.

[86] Li J (2006). Dkk1-mediated inhibition of wnt signaling in bone results in osteopenia. Bone.

[87] MacDonald BT (2007). Bone mass is inversely proportional to Dkk1 levels in mice. Bone.

[88] Jaschke NP (2022). Circulating Dickkopf1 parallels metabolic adaptations and predicts Disease trajectories in patients with COVID-19. J Clin Endocrinol Metab.

[89] Brunkow ME, Gardner JC, Van Ness J, Paeper BW, Kovacevich BR, Proll S, Skonier JE, Zhao L, Sabo PJ, Fu Y, Alisch RS, Gillett L, Colbert T, Tacconi P, Galas D, Hamersma H, Beighton P, Mulligan J. Bone dysplasia sclerosteosis results from loss of the SOST gene product, a novel cystine knot-containing protein. Am J Hum Genet. 2001 Mar;68(3):577–89. 10.1086/318811 PMC1274471 11179006

[90] Balemans W (2001). Increased bone density in sclerosteosis is due to the deficiency of a novel secreted protein (SOST). Hum Mol Genet.

[91] Balemans W (2002). Identification of a 52 kb deletion downstream of the < em > SOST gene in patients with Van Buchem disease. J Med Genet.

[92] Kim SJ (2011). Identification of signal peptide domain SOST mutations in autosomal dominant craniodiaphyseal dysplasia. Hum Genet.

[93] Koch A (2017). Relevance of serum sclerostin concentrations in critically ill patients. J Crit Care.

[94] Tirandi A et al. (2023) Early sclerostin assessment in frail elderly patients with sepsis: insights on short- and long-term mortality prediction. Intern Emerg Med. 10.1007/s11739-023-03223-w PMC10412666 36943596

[95] Yuen M, Ottenheijm CAC (2020). Nebulin: big protein with big responsibilities. J Muscle Res Cell Motil.

[96] Soares AJ, Santos MF, Chung J, David CM, Domont GB (2007). [Proteomics and sepsis: new perspectives for diagnosis]. Rev Bras Ter Intensiva.

[97] Wu EY, Campbell MJ (2021). Cardiac manifestations of Multisystem Inflammatory Syndrome in Children (MIS-C) following COVID-19. Curr Cardiol Rep.

[98] Sperotto F (2021). Cardiac manifestations in SARS-CoV-2-associated multisystem inflammatory syndrome in children: a comprehensive review and proposed clinical approach. Eur J Pediatr.

[99] Brindle NPJ, Saharinen P, Alitalo K (2006). Signaling and functions of Angiopoietin-1 in Vascular Protection. Circul Res.

[100] Patel MA (2022). Elevated vascular transformation blood biomarkers in Long-COVID indicate angiogenesis as a key pathophysiological mechanism. Mol Med.

[101] Wismans LV (2023). Increase of mast cells in COVID-19 pneumonia may contribute to pulmonary fibrosis and thrombosis. Histopathology.

[102] Yatim N (2021). Platelet activation in critically ill COVID-19 patients. Ann Intensive Care.

[103] Risau W (1992). Platelet-derived growth factor is angiogenic in vivo. Growth Factors.

[104] Talarico LB, et al. Distinct Immune phenotypes and cytokine profiles in children with Differing Severity of COVID-19. Pediatr Infect Dis J. 2022;41:919–26. 10.1097/INF.0000000000003669 PMC9555606 36102684

[105] Wang H, Wang H, Cheng P. Kawasaki disease on PDGF expression and VSMC proliferation. J Tongji Med Univ. 1998;18:243–6. 10.1007/BF02886483 10806856

[106] Ning Z, Liu K, Xiong H (2021). Roles of BTLA in Immunity and Immune disorders. Front Immunol.

[107] Shubin NJ, Monaghan SF, Heffernan DS, Chung C-S, Ayala A (2013). B and T lymphocyte attenuator expression on CD4 + T-cells associates with sepsis and subsequent infections in ICU patients. Crit Care.

[108] Surolia I (2010). Functionally defective germline variants of sialic acid acetylesterase in autoimmunity. Nature.

[109] Cariappa A (2009). B cell antigen receptor signal strength and peripheral B cell development are regulated by a 9-O-acetyl sialic acid esterase. J Exp Med.

[110] Yang CA, Huang YL, Chiang BL (2022). Innate immune response analysis in COVID-19 and kawasaki disease reveals MIS-C predictors. J Formos Med Assoc.

[111] Umar S (2022). Inhibition of IRAK4 dysregulates SARS-CoV-2 spike protein-induced macrophage inflammatory and glycolytic reprogramming. Cell Mol Life Sci.

[112] Arevalo JA, Vazquez-Medina JP. (2018) The role of Peroxiredoxin 6 in Cell Signaling. Antioxid (Basel) 7. 10.3390/antiox7120172 PMC6316032 30477202

[113] Davies MJ, Hawkins CL (2020). The role of Myeloperoxidase in Biomolecule Modification, chronic inflammation, and Disease. Antioxid Redox Signal.

[114] Santos-Rebouças CB (2022). Host genetic susceptibility underlying SARS-CoV-2-associated multisystem inflammatory syndrome in Brazilian children. Mol Med.

[115] Li Y et al. (2021) Bioinformatics Analysis for Identifying Pertinent Pathways and Genes in Sepsis. *Comput Math Methods Med* 2021: 2085173. 10.1155/2021/2085173 PMC8575597 34760021

[116] Caldarale F (2021). Plasmacytoid dendritic cells depletion and elevation of IFN-γ dependent chemokines CXCL9 and CXCL10 in Children with Multisystem Inflammatory Syndrome. Front Immunol.

[117] Moratto D (2020). Immune response in children with COVID-19 is characterized by lower levels of T-cell activation than infected adults. Eur J Immunol.

[118] Callahan V et al. (2021) The Pro-Inflammatory Chemokines CXCL9, CXCL10 and CXCL11 Are Upregulated Following SARS-CoV-2 Infection in an AKT-Dependent Manner. *Viruses* 13. 10.3390/v13061062 PMC8226769 34205098

[119] Cheng W, Hornung R, Xu K, Yang C, Li J (2021). Complement C3 identified as a unique risk factor for disease severity among young COVID-19 patients in Wuhan, China. Sci Rep.

