# Supplementary material for: The plasma proteome differentiates the multisystem inflammatory syndrome in children (MIS-C) from children with SARS-CoV-2 negative sepsis
Source: Mol Med. 2024 Apr 17;30:51. doi: 10.1186/s10020-024-00806-x (PMC11022403; doi:10.1186/s10020-024-00806-x)
Supplement: Supplementary file 1 — Supplementary Material 1 [file 10020_2024_806_MOESM1_ESM.docx]

**Supplemental Table 1. Importance of the 58 proteins in Comparing MIS-C and SCNS patient proteomes on PICU Day 1, as well as Healthy Controls subjects.**

| **Assay** | **MIS-C** | **SCNS** | **Controls** | **P Value** |
| --- | --- | --- | --- | --- |
| KLK13 | 0.7 (0.6-0.9) | 2.9 (1.9-3.4) | 3.2 (2.8-3.5) | <0.005 |
| LTA4H | 0.8 (0.3-1.9) | 0.1 (0.0-0.1) | 0.0 (0.0-0.0) | <0.005 |
| BTLA | 0.4 (0.2-0.6) | 1.9 (1.6-2.4) | 1.3 (1.2-1.4) | <0.005 |
| IRAK4 | 7.7 (4.8-12.3) | 1.7 (1.1-2.1) | 0.8 (0.5-1.3) | <0.005 |
| PRDX6 | 6.5 (3.5-11.5) | 0.9 (0.6-1.7) | 0.6 (0.5-0.8) | <0.005 |
| CREBZF | 0.1 (0.0-0.2) | 1.0 (0.8-1.1) | 0.7 (0.7-0.8) | <0.005 |
| DKK1 | 5.7 (3.6-7.3) | 0.9 (0.6-0.9) | 0.9 (0.9-1.2) | <0.005 |
| C3 | 1.1 (0.6-1.4) | 0.2 (0.2-0.2) | 0.2 (0.2-0.2) | <0.005 |
| SAMD9L | 30.9 (18.4-54.6) | 3.0 (2.0-4.3) | 1.1 (0.8-1.9) | <0.005 |
| ANGPT1 | 5.7 (3.5-6.5) | 0.5 (0.4-0.6) | 0.4 (0.3-0.6) | <0.005 |
| CXCL11 | 217.1 (65.5-285.7) | 1.3 (0.7-5.4) | 0.7 (0.6-1.0) | <0.005 |
| PPBP | 7.8 (4.8-10.1) | 0.2 (0.1-0.3) | 0.3 (0.1-0.5) | <0.005 |
| BMP4 | 0.0 (0.0-0.0) | 0.1 (0.1-0.1) | 0.1 (0.1-0.1) | <0.005 |
| SOST | 2.6 (2.0-5.7) | 0.7 (0.5-0.9) | 1.1 (1.0-1.4) | <0.005 |
| PDGFA | 4.7 (3.6-6.3) | 0.5 (0.4-0.5) | 0.5 (0.4-0.6) | <0.005 |
| NEB | 0.8 (0.6-1.2) | 3.6 (3.2-4.4) | 3.2 (2.8-3.8) | <0.005 |
| VAMP8 | 18.8 (13.6-28.9) | 2.7 (2.5-5.1) | 1.8 (1.1-3.6) | <0.005 |
| SIAE | 2.4 (1.7-2.9) | 0.8 (0.7-0.9) | 0.7 (0.6-0.9) | <0.005 |
| SERPINA1 | 1.9 (1.8-2.0) | 1.2 (1.1-1.3) | 0.8 (0.8-1.0) | <0.005 |
| PRTG | 0.8 (0.7-1.0) | 1.6 (1.4-2.1) | 1.6 (1.5-1.7) | <0.005 |
| MRPL58 | 0.7 (0.5-0.8) | 1.7 (1.4-1.8) | 1.3 (1.2-2.1) | <0.005 |
| AZU1 | 45.6 (38.9-51.0) | 2.0 (1.5-2.4) | 0.5 (0.5-0.6) | <0.005 |
| CASP8 | 10.2 (5.5-16.5) | 0.5 (0.3-0.6) | 0.2 (0.2-0.3) | <0.005 |
| HLA-DRA | 3.9 (3.1-5.5) | 0.9 (0.8-1.2) | 0.9 (0.8-1.2) | <0.005 |
| MPO | 14.3 (12.6-14.8) | 2.2 (1.6-2.6) | 0.8 (0.7-0.9) | <0.005 |
| CCL5 | 6.0 (2.2-8.4) | 0.3 (0.2-0.4) | 0.5 (0.1-0.7) | <0.005 |
| PTN | 0.2 (0.1-0.5) | 3.0 (1.6-5.0) | 1.8 (1.4-2.4) | <0.005 |
| ABHD14B | 6.0 (4.5-7.4) | 0.6 (0.3-1.0) | 0.4 (0.3-0.4) | <0.005 |
| ARG1 | 3.5 (2.4-3.9) | 0.6 (0.5-0.9) | 0.3 (0.3-0.5) | <0.005 |
| GBP2 | 19.3 (13.6-32.8) | 1.5 (0.7-2.3) | 0.4 (0.4-0.5) | <0.005 |
| DCTN1 | 10.3 (8.0-12.9) | 1.8 (1.3-2.2) | 1.4 (1.0-1.8) | <0.005 |
| PDGFB | 6.5 (4.2-10.0) | 0.2 (0.2-0.3) | 0.3 (0.2-0.9) | <0.005 |
| ATP6V1F | 3.5 (2.1-4.5) | 0.8 (0.4-1.0) | 0.5 (0.4-0.9) | <0.005 |
| GLI2 | 0.7 (0.6-0.8) | 1.5 (1.2-1.8) | 1.3 (1.1-1.5) | <0.005 |
| ENO1 | 11.7 (6.3-14.9) | 1.6 (1.2-2.2) | 1.1 (0.9-1.8) | <0.005 |
| SPARC | 6.2 (4.3-7.7) | 0.7 (0.5-0.9) | 1.4 (1.2-1.8) | <0.005 |
| SCRN1 | 6.3 (3.6-10.8) | 0.8 (0.6-1.1) | 0.6 (0.5-0.7) | <0.005 |
| CLEC1B | 11.2 (9.1-18.3) | 1.7 (1.4-1.9) | 1.0 (0.9-2.7) | <0.005 |
| SORT1 | 2.8 (2.0-3.0) | 0.9 (0.7-1.0) | 0.8 (0.7-1.0) | <0.005 |
| PSMD1 | 6.2 (3.1-7.1) | 0.8 (0.6-1.0) | 0.4 (0.2-0.7) | <0.005 |
| RNASE3 | 143.1 (47.0-172.4) | 6.1 (3.7-7.6) | 0.7 (0.5-0.8) | <0.005 |
| HPSE | 13.4 (10.3-17.6) | 1.2 (1.0-1.5) | 1.2 (0.9-2.6) | <0.005 |
| DCTN2 | 18.0 (9.9-45.0) | 1.2 (0.9-1.5) | 0.9 (0.7-1.3) | <0.005 |
| KIAA1549L | 0.0 (0.0-0.7) | 3.3 (2.5-3.8) | 2.5 (1.7-3.7) | <0.005 |
| CACYBP | 23.0 (12.5-38.7) | 2.1 (1.8-2.6) | 1.2 (0.8-2.9) | <0.005 |
| ITIH4 | 1.7 (1.5-2.3) | 0.8 (0.7-0.8) | 0.8 (0.7-1.0) | <0.005 |
| ATP6V1G1 | 12.0 (7.6-18.8) | 1.1 (0.9-1.6) | 0.8 (0.6-1.1) | <0.005 |
| STAT5B | 16.1 (8.9-19.9) | 1.5 (1.2-1.8) | 0.7 (0.5-0.7) | <0.005 |
| BDNF | 2.0 (0.9-2.6) | 0.1 (0.1-0.2) | 0.1 (0.1-0.4) | <0.005 |
| EDF1 | 10.3 (3.9-15.7) | 0.8 (0.5-1.4) | 0.6 (0.4-1.5) | <0.005 |
| CA4 | 1.9 (1.7-2.3) | 0.9 (0.8-1.0) | 1.1 (1.0-1.2) | <0.005 |
| FCN1 | 1.5 (1.3-1.9) | 0.2 (0.2-0.3) | 0.5 (0.3-0.5) | <0.005 |
| EGFL7 | 1.7 (1.2-2.3) | 4.8 (4.5-6.7) | 3.9 (3.5-4.6) | <0.005 |
| SERPINI1 | 1.0 (0.9-1.2) | 0.4 (0.3-0.6) | 0.7 (0.6-0.8) | <0.005 |
| F10 | 1.1 (1.1-1.4) | 0.6 (0.5-0.8) | 0.9 (0.9-1.0) | <0.005 |
| EGF | 13.9 (6.2-32.4) | 0.4 (0.2-0.5) | 0.4 (0.3-1.1) | <0.005 |
| LYSMD3 | 0.6 (0.5-0.8) | 0.1 (0.1-0.3) | 0.2 (0.1-0.4) | <0.005 |
| ATP6V1D | 0.8 (0.7-0.9) | 1.1 (1.0-1.2) | 1.0 (0.9-1.2) | <0.005 |

**Note:** The P Value represents MIS-C versus SCNS versus healthy controls. P Value is FDR Adjusted Kruskal-Wallis Test.

**Supplemental Table 2. Importance of the 58 proteins in Comparing MIS-C and SCNS patient proteomes on PICU Day 1.**

| **Rank** | **Assay** | **MIS-C**  **Patients** | **SCNS**  **Patients** | **P Value** | **ROC AUC** | **F1** | **Importance** |
| --- | --- | --- | --- | --- | --- | --- | --- |
| 1 | KLK13 | 0.7 (0.6-0.9) | 2.9 (1.9-3.4) | <0.005 | 1.00 | 0.95 | 5.02 |
| 2 | LTA4H | 0.8 (0.3-1.9) | 0.1 (0.0-0.1) | <0.005 | 1.00 | 0.75 | 5.01 |
| 3 | BTLA | 0.4 (0.2-0.6) | 1.9 (1.6-2.4) | <0.005 | 1.00 | 0.97 | 4.87 |
| 4 | IRAK4 | 7.7 (4.8-12.3) | 1.7 (1.1-2.1) | <0.005 | 1.00 | 0.95 | 4.78 |
| 5 | PRDX6 | 6.5 (3.5-11.5) | 0.9 (0.6-1.7) | <0.005 | 1.00 | 0.93 | 4.77 |
| 6 | CREBZF | 0.1 (0.0-0.2) | 1.0 (0.8-1.1) | <0.005 | 1.00 | 0.96 | 4.77 |
| 7 | DKK1 | 5.7 (3.6-7.3) | 0.9 (0.6-0.9) | <0.005 | 1.00 | 0.97 | 4.64 |
| 8 | C3 | 1.1 (0.6-1.4) | 0.2 (0.2-0.2) | <0.005 | 1.00 | 0.82 | 4.56 |
| 9 | SAMD9L | 30.9 (18.4-54.6) | 3.0 (2.0-4.3) | <0.005 | 0.99 | 0.93 | 2.66 |
| 10 | ANGPT1 | 5.7 (3.5-6.5) | 0.5 (0.4-0.6) | <0.005 | 1.00 | 0.95 | 2.64 |
| 11 | CXCL11 | 217.1 (65.5-285.7) | 1.3 (0.7-5.4) | <0.005 | 0.99 | 0.93 | 2.50 |
| 12 | PPBP | 7.8 (4.8-10.1) | 0.2 (0.1-0.3) | <0.005 | 1.00 | 0.94 | 2.40 |
| 13 | BMP4 | 0.0 (0.0-0.0) | 0.1 (0.1-0.1) | <0.005 | 0.99 | 0.94 | 2.40 |
| 14 | SOST | 2.6 (2.0-5.7) | 0.7 (0.5-0.9) | <0.005 | 1.00 | 0.94 | 2.38 |
| 15 | PDGFA | 4.7 (3.6-6.3) | 0.5 (0.4-0.5) | <0.005 | 1.00 | 0.92 | 2.22 |
| 16 | NEB | 0.8 (0.6-1.2) | 3.6 (3.2-4.4) | <0.005 | 1.00 | 0.99 | 2.21 |
| 17 | VAMP8 | 18.8 (13.6-28.9) | 2.7 (2.5-5.1) | <0.005 | 0.99 | 0.94 | 2.13 |
| 18 | SIAE | 2.4 (1.7-2.9) | 0.8 (0.7-0.9) | <0.005 | 0.99 | 0.85 | 1.90 |
| 19 | SERPINA1 | 1.9 (1.8-2.0) | 1.2 (1.1-1.3) | <0.005 | 0.98 | 0.92 | 1.82 |
| 20 | PRTG | 0.8 (0.7-1.0) | 1.6 (1.4-2.1) | <0.005 | 0.97 | 0.87 | 1.57 |
| 21 | MRPL58 | 0.7 (0.5-0.8) | 1.7 (1.4-1.8) | <0.005 | 1.00 | 0.93 | 1.46 |
| 22 | AZU1 | 45.6 (38.9-51.0) | 2.0 (1.5-2.4) | <0.005 | 0.99 | 0.93 | 1.46 |
| 23 | CASP8 | 10.2 (5.5-16.5) | 0.5 (0.3-0.6) | <0.005 | 0.99 | 0.90 | 1.44 |
| 24 | HLA-DRA | 3.9 (3.1-5.5) | 0.9 (0.8-1.2) | <0.005 | 0.98 | 0.87 | 1.43 |
| 25 | MPO | 14.3 (12.6-14.8) | 2.2 (1.6-2.6) | <0.005 | 0.99 | 0.92 | 1.42 |
| 26 | CCL5 | 6.0 (2.2-8.4) | 0.3 (0.2-0.4) | <0.005 | 1.00 | 0.89 | 1.32 |
| 27 | PTN | 0.2 (0.1-0.5) | 3.0 (1.6-5.0) | <0.005 | 0.99 | 0.90 | 1.31 |
| 28 | ABHD14B | 6.0 (4.5-7.4) | 0.6 (0.3-1.0) | <0.005 | 0.98 | 0.90 | 1.29 |
| 29 | ARG1 | 3.5 (2.4-3.9) | 0.6 (0.5-0.9) | <0.005 | 0.98 | 0.90 | 1.28 |
| 30 | GBP2 | 19.3 (13.6-32.8) | 1.5 (0.7-2.3) | <0.005 | 0.98 | 0.88 | 1.25 |
| 31 | DCTN1 | 10.3 (8.0-12.9) | 1.8 (1.3-2.2) | <0.005 | 0.98 | 0.92 | 1.24 |
| 32 | PDGFB | 6.5 (4.2-10.0) | 0.2 (0.2-0.3) | <0.005 | 0.99 | 0.91 | 1.19 |
| 33 | ATP6V1F | 3.5 (2.1-4.5) | 0.8 (0.4-1.0) | <0.005 | 0.99 | 0.88 | 1.18 |
| 34 | GLI2 | 0.7 (0.6-0.8) | 1.5 (1.2-1.8) | <0.005 | 0.98 | 0.89 | 1.16 |
| 35 | ENO1 | 11.7 (6.3-14.9) | 1.6 (1.2-2.2) | <0.005 | 0.98 | 0.92 | 1.15 |
| 36 | SPARC | 6.2 (4.3-7.7) | 0.7 (0.5-0.9) | <0.005 | 0.99 | 0.94 | 1.06 |
| 37 | SCRN1 | 6.3 (3.6-10.8) | 0.8 (0.6-1.1) | <0.005 | 0.99 | 0.87 | 0.98 |
| 38 | CLEC1B | 11.2 (9.1-18.3) | 1.7 (1.4-1.9) | <0.005 | 0.98 | 0.95 | 0.98 |
| 39 | SORT1 | 2.8 (2.0-3.0) | 0.9 (0.7-1.0) | <0.005 | 0.98 | 0.92 | 0.97 |
| 40 | PSMD1 | 6.2 (3.1-7.1) | 0.8 (0.6-1.0) | <0.005 | 0.97 | 0.89 | 0.95 |
| 41 | RNASE3 | 143.1 (47.0-172.4) | 6.1 (3.7-7.6) | <0.005 | 0.98 | 0.89 | 0.94 |
| 42 | HPSE | 13.4 (10.3-17.6) | 1.2 (1.0-1.5) | <0.005 | 0.99 | 0.95 | 0.94 |
| 43 | DCTN2 | 18.0 (9.9-45.0) | 1.2 (0.9-1.5) | <0.005 | 0.98 | 0.94 | 0.92 |
| 44 | KIAA1549L | 0.0 (0.0-0.7) | 3.3 (2.5-3.8) | <0.005 | 0.99 | 0.94 | 0.92 |
| 45 | CACYBP | 23.0 (12.5-38.7) | 2.1 (1.8-2.6) | <0.005 | 0.99 | 0.93 | 0.89 |
| 46 | ITIH4 | 1.7 (1.5-2.3) | 0.8 (0.7-0.8) | <0.005 | 0.97 | 0.87 | 0.89 |
| 47 | ATP6V1G1 | 12.0 (7.6-18.8) | 1.1 (0.9-1.6) | <0.005 | 0.98 | 0.91 | 0.87 |
| 48 | STAT5B | 16.1 (8.9-19.9) | 1.5 (1.2-1.8) | <0.005 | 0.97 | 0.89 | 0.70 |
| 49 | BDNF | 2.0 (0.9-2.6) | 0.1 (0.1-0.2) | <0.005 | 0.99 | 0.83 | 0.65 |
| 50 | EDF1 | 10.3 (3.9-15.7) | 0.8 (0.5-1.4) | <0.005 | 0.97 | 0.90 | 0.56 |
| 51 | CA4 | 1.9 (1.7-2.3) | 0.9 (0.8-1.0) | <0.005 | 0.97 | 0.89 | 0.50 |
| 52 | FCN1 | 1.5 (1.3-1.9) | 0.2 (0.2-0.3) | <0.005 | 0.98 | 0.86 | 0.50 |
| 53 | EGFL7 | 1.7 (1.2-2.3) | 4.8 (4.5-6.7) | <0.005 | 0.98 | 0.90 | 0.30 |
| 54 | SERPINI1 | 1.0 (0.9-1.2) | 0.4 (0.3-0.6) | <0.005 | 0.99 | 0.90 | 0.29 |
| 55 | F10 | 1.1 (1.1-1.4) | 0.6 (0.5-0.8) | <0.005 | 1.00 | 0.93 | 0.16 |
| 56 | EGF | 13.9 (6.2-32.4) | 0.4 (0.2-0.5) | <0.005 | 0.97 | 0.87 | 0.12 |
| 57 | LYSMD3 | 0.6 (0.5-0.8) | 0.1 (0.1-0.3) | <0.005 | 0.97 | 0.87 | 0.06 |
| 58 | ATP6V1D | 0.8 (0.7-0.9) | 1.1 (1.0-1.2) | <0.005 | 0.97 | 0.84 | 0.02 |

**Note:** The P Value represents MIS-C versus SCNS. P Value is FDR Adjusted Mann-Whitney U Test.

**Supplemental Table 3. Function of the 58 Identified Proteins.**

| **Rank** | **UniProt** | **Assay** | **Function** |
| --- | --- | --- | --- |
| 1 | Q9UKR3 | KLK13 | Kallikrein 13 (KLK13), a serine protease, is primarily associated with cancer and linked to poor outcomes (1-3). It has also been identified as a priming protease for the human coronavirus HKU1 (HCoV-HKU1) and mediates entry through respiratory epithelial cells (4). |
| 2 | P09960 | LTA4H | Leukotriene A4 Hydrolase (LTA4H) catalyzes the production of leukotriene B4, an immune system modulator (5). The enzyme is a potential anti-inflammatory agent and cancer target (6, 7). |
| 3 | Q7Z6A9 | BTLA | B and T lymphocyte attenuator (BTLA) is a CD28 superfamily member that is important in inhibitory cosignaling and is detected by most lymphocytes (8). It has been previously identified to be upregulated in CD4+ and CD8+ cells of COVID-19 patients (9-11). Similarly, critically ill sepsis patients also show increased BTLA expression (12). |
| 4 | Q9NWZ3 | IRAK4 | Interleukin 1 receptor-associated kinase 4 (IRAK4) is a critical component of the IL-1/TLR signalling pathway involved in innate immunity (13). IRAK4 deficiencies have been linked to decreased innate immunity to bacterial and viral pathogens (14). IRAK4 is identified to be upregulated in COVID-19 patients and proposed as a potential knockdown therapy target to decrease inflammation (15, 16). |
| 5 | P30041 | PRDX6 | Peroxiredoxin-6 (PRDX6) is an antioxidant enzyme that is expressed in all organs and cell types (17). It repairs peroxidised cell membranes and overexpression has been identified to be linked to growth-promoting in cancer tumors (18, 19). |
| 6 | Q9NS37 | CREBZF | CREB/ATF bZIP transcription factor (CREBZF) is expressed in the liver, kidney, and pancreas and linked to innate immunity and cancer modulation (20-22). |
| 7 | O94907 | DKK1 | Dickkopf-1 (DKK1) is a WNT antagonist and functions by binding to low-density lipoprotein receptor-related protein 6 (LRP6), a necessary WNT activation co-receptor (23). DKK1 is also a downstream target of bone morphogenetic proteins and involved in adult bone formation (24). |
| 8 | P01024 | C3 | Complement 3 (C3) is a critical activation component of all complement innate immunity pathways (25). |
| 9 | Q8IVG5 | SAMD9L | Sterile Alpha Motif Domain Containing 9 Like (SAMD9L) is paralogous to IFN genes and involved in decreasing viral replication (26). SNPs in SAMD9L have been proposed to decrease IVIG response in those affected by Kawasaki disease (27). |
| 10 | Q15389 | ANGPT1 | Angiopoietin-1 (ANGPT1), part of the angiopoietin family, is expressed in vascular smooth muscle cells, induces TIE2 tyrosine kinase and activates angiogenesis and vascular protective effects including suppressing plasma leakage, inhibiting vascular inflammation, preventing endothelial death, and enlargement of existing vessels (28-30). |
| 11 | O14625 | CXCL11 | C-X-C motif chemokine 11 (CXCL11) is a part of the CXC chemokine family with widespread expression (31). CXCL11 interacts with CXCR receptors, primarily with CXCR3 with high affinity, for the localization of T cells (31). |
| 12 | P02775 | PPBP | Pro-Platelet Basic Protein (PPBP) or CXC motif ligand 7 (CXCL7) is a platelet-derived growth factor (32). Primarily increases thrombosis response but also stimulates various cell growth processes (33, 34). |
| 13 | P12644 | BMP4 | Bone morphogenetic protein 4 (BMP4) is part of the TGF-beta family and involved in bone and cartilage development (35). It is also involved in pro-angiogenesis and vascular transformation (36). |
| 14 | Q9BQB4 | SOST | Sclerostin (SOST) is expressed primarily in osteocytes and binds to LRP6 co-receptor for WNT activation and acts as a WNT inhibitor and negative regulator of bone formation (37, 38). Downregulation of SOST expression can lead to high bone mass malignancies including Van Buchem disease and Sclerosteosis (39-41). |
| 15 | P04085 | PDGFA | Platelet-Derived Growth Factor (PDGF) subunit A (PDGFA) is one of the four members of the PDGF family (42). Binding with PDGFB, they activate PDGF receptors and enable cell proliferation, migration, and survival as well as vascular transformation (43). |
| 16 | P20929 | NEB | Nebulin (NEB) is a large actin-binding muscle protein expressed in the skeletal muscle with some mouse model evidence of heart expression (44). It maintains structural integrity of the sarcomeres and regulates muscle contraction (45). |
| 17 | Q9BV40 | VAMP8 | Vesicle-associated membrane protein 8 (VAMP8) is part of a soluble N-ethylmaleimide-sensitive factor attachment protein receptors (SNAREs) subfamily. It functions to fuse synaptic vesicles with the presynaptic membrane, primarily in autophagy and for granule secretion in platelets (46-48). |
| 18 | Q9HAT2 | SIAE | Sialic acid acetylesterase (SIAE) removes the 9-O-acetylation from sialic acids and decreases B lymphocyte antigen receptor signalling in mice (49). Mutations resulting in decreased functional expression of SIAE are associated with autoimmunity disorders and possible coronavirus receptors (50-52). |
| 19 | P01009 | SERPINA1 | Alpha-1-antitrypsin (SERPINA1) is part of the Serpin family and is an inhibitor of serine proteases including elastase, trypsin, and thrombin (53). Deficiency in SERPINA1 can lead to COPD or cryptogenic liver disease (54). |
| 20 | Q2VWP7 | PRTG | Protogenin (PRTG) is a member of the immunoglobulin superfamily primarily identified as a development protein in animal models (55, 56). |
| 21 | Q14197 | MRPL58 | Mitochondrial Ribosomal Protein L58 (MRPL58) is a peptidyl-tRNA hydrolase and the release factor for the large mitochondrial ribosome. MRPL58 primarily hydrolysis prematurely terminated peptidyl-tRNA (57) and is important for cell survival (58). |
| 22 | P20160 | AZU1 | Azurocidin (AZU1) is a chemotactic glycoprotein released by neutrophils in response to Gram-negative bacteria. While it primarily attracts monocytes and macrophages in bacterial infections, it also alters vascular permeability (59, 60). |
| 23 | Q14790 | CASP8 | Caspase-8 (CASP8) is a cysteine-aspartic acid protease family member that is critical in programmed cell death (61). Other non-apoptotic functions for CASP8 include cell migration, adhesion, monocyte differentiation, and T-cell activation (62, 63). |
| 24 | P01903 | HLA-DRA | Major Histocompatibility Complex, Class II, DR Alpha (HLA-DRA) is an alpha chain HLA class II (MHCII) expressed on antigen-presenting cells and critical for the removal of infectious agents (64). |
| 25 | P05164 | MPO | Myeloperoxidase (MPO) is expressed by polymorphonuclear leukocytes and is a peroxidase enzyme. The hypochlorous acid produced by the catalyzed reaction aids in immunity by causing oxidative damage to infectious agents (65, 66). |
| 26 | P13501 | CCL5 | C-C motif ligand 5 (CCL5 a.k.a RANTES) is a chemokine that is expressed by T-cells and monocytes and attracts T cells, eosinophils, and basophils (67). |
| 27 | P21246 | PTN | Pleiotrophin (PTN) or neurite growth-promoting factor 1 (NEGF1) is a heparin-binding protein expressed in the brain (68). It is also involved in bone development and remodelling by interacting with osteoblasts as well as angiogenesis (69, 70). |
| 28 | Q96IU4 | ABHD14B | Abhydrolase Domain Containing 14B (ABHD14B) is a lysine deacetylase that is proposed to impact glucose metabolism (71, 72). |
| 29 | P05089 | ARG1 | Arginase 1 (ARG1) is expressed mainly in the liver and is part of the urea cycle hydrolysis of arginine to ornithine and urea (73). ARG1 deficiency causes a rare liver autosomal recessive disorder (74). ARG1 also helps manage arginine metabolism which is important for inflammatory response (75). |
| 30 | P32456 | GBP2 | Guanylate Binding Protein 2 (GBP2) is induced by interferons and hydrolyzes GTP into GMP and GDP (76). GBP2 also participates in pathogen defence including antiviral activity for viral pathogens with envelopes (77). |
| 31 | Q14203 | DCTN1 | Dynactin subunit 1 (DCTN1) is part of the dynactin complex which acts as a dynein regulator and tether (78, 79). DCTN1 mutations and dysregulation has been implicated in various neurological disorders including ALS, multiple sclerosis, and Perry syndrome (80-82). |
| 32 | P01127 | PDGFB | Platelet-Derived Growth Factor (PDGF) subunit B (PDGFB) is one of the four members of the PDGF family (42). Along with PDGFB’s mesenchymal mitogenic functions, it is involved in angiogenesis and hematopoiesis (83). |
| 33 | Q16864 | ATP6V1F | ATPase H+ Transporting V1 Subunit F (ATP6V1F) is a part of vacuolar ATPase (V-ATPase) responsible for organelle acidification (84). |
| 34 | P10070 | GLI2 | GLI Family Zinc Finger 2 (GLI2) is a transcription regulator and is primarily identified to be involved in the Sonic Hedgehog signally pathway, part of the vertebrate embryonic development (85). |
| 35 | P06733 | ENO1 | Enolase 1 (ENO1) is a glycolytic enzyme which catalyzes the inter-conversion of 2-phosphoglycerate to phosphoenolpyruvate. ENO1 also functions to stabilize mitochondrial membrane and is also linked to possible cancers (86, 87) |
| 36 | P09486 | SPARC | Secreted Protein Acidic and Cysteine Rich (SPARC) is a matrix-associated protein expressed and secreted by osteoblasts that binds to Ca+2 and aids in bone mineralization and collagen binding (88). SPARC is also expressed by other cell types and involved in MMP activity, cell attachment and proliferation (89). |
| 37 | Q12765 | SCRN1 | Secernin 1 (SCRN1) is a member of the secerning family of proteins and is involved in regulating exocytosis in MAST cells (90). SCRN1 is a potential biomarker for various cancers and also may be involved in mouse development (91, 92). |
| 38 | Q9P126 | CLEC1B | C-Type Lectin Domain Family 1 Member B (CLEC1B) is expressed by myeloid cells and Natural Killer cells, interacting with MHCI molecules (93). It is also found on platelets and activates platelets through the syk-dependent mechanism (94). |
| 39 | Q99523 | SORT1 | Sortilin 1 (SORT1), part of the VPS10-related sortilin family, is expressed in various cell types and organs. SORT1 is involved in the Golgi as a sorting receptor. Dysregulation impacts lipid metabolism and is involved in metabolic and cardiovascular diseases (95, 96). |
| 40 | Q99460 | PSMD1 | Proteasome 26S Subunit, Non-ATPase 1 (PSMD1) is part of the 26S proteasome which degrades intracellular proteins to maintain homeostasis (97). |
| 41 | P12724 | RNASE3 | Ribonuclease A Family Member 3 (RNASE3) gene encodes the eosinophil cationic protein (ECP3) released in eosinophil granules and acts as a host defence protein (98, 99). |
| 42 | Q9Y251 | HPSE | Herparanase (HPSE) is an endoglycosidase expressed primarily in placental trophoblasts, blood-borne cells, and keratinocytes that cleaves herpan sulfate proteoglycans found on cell surfaces and the extracellular matrix. This facilitates cell migration and proliferation as well as signalling. (100, 101) |
| 43 | Q13561 | DCTN2 | Dynactin subunit 2 (DCTN2) also referred to as dynamitin is part of the dynactin complex however it operates as an inhibitor. DCTN2 overexpression disrupts the dynactin complex by disassembling it, including DCTN1 (102, 103). |
| 44 | Q6ZVL6 | KIAA1549L | KIAA1549 Like (KIAA1549L) is associated as a potential cancer biomarker however, there is limited literature (104). |
| 45 | Q9HB71 | CACYBP | Calcyclin Binding Protein (CACYBP) is expressed in various mammal tissues with higher expression in neurons and is involved in homeostasis in cancers via ubiquitinoylation (105). |
| 46 | Q14624 | ITIH4 | Inter-Alpha-Trypsin Inhibitor Heavy Chain 4 (ITIH4) is expressed in the liver and secreted into blood (106). ITIH4 has been implicated in inflammation, organ injury, epithelial adhesion and migration (107). |
| 47 | O75348 | ATP6V1G1 | ATPase H+ Transporting V1 Subunit G1 (ATP6V1G1) is a part of vacuolar ATPase (V-ATPase) responsible for organelle acidification (84). |
| 48 | P51692 | STAT5B | Signal Transducer and Activator Of Transcription 5B (STAT5B) is a transcription activator that can be activated by pathogens and other cytokines (108, 109). It is also involved in signal transduction via growth factors and cytokines (110, 111). |
| 49 | P23560 | BDNF | Brain-Derived Neurotrophic Factor (BDNF) is part of the nerve growth factor family and binds to TrkB and low-affinity nerve growth receptor (LNGFR) expressed on neuron cell surfaces (112, 113). Brain-derived neurotrophic factor (BDNF) is a critical synaptic protein in the brain associated with neuronal survival, plasticity and signalling as well as memory, learning, depression, and anxiety (114-116) |
| 50 | O60869 | EDF1 | Endothelial Differentiation Related Factor 1 (EDF1) is involved in angiogenesis via endothelial cell differentiation and is upregulated in cell proliferation (117). It is also possibly involved in regulating nitric oxide release in response to VEGF during angiogenesis (118, 119). |
| 51 | P22748 | CA4 | Carbonic Anhydrase 4 (CA4) is a zinc metalloenzyme that maintains pH by catalyzing the hydration of carbon dioxide into bicarbonate. Mutations in CA4 have been associated with retinal disorders (120, 121). |
| 52 | O00602 | FCN1 | Ficolin 1 (FCN1), expressed primarily in blood leukocytes and bone marrow plays a role in innate immunity by activating the lectin complement after binding sugar or acetylated pathogen-associated molecular patterns (122). FCN1 mutations and is involved in various inflammatory and immune disorders (123-125). |
| 53 | Q9UHF1 | EGFL7 | EGF Like Domain Multiple 7 (EGFL7) is expressed in endothelial cells and involved in angiogenesis and vascular transformation, including during inflammatory conditions (126-128). EGFL7 may inhibit PDGFB-mediated cell migration, however, proliferation is still active (129). |
| 54 | Q99574 | SERPINI1 | Serpin Family I Member 1 (SERPINI1) is a serine proteinase inhibitor that is involved in the adult nervous system. It selectively inhibits tissue plasminogen activator and is involved in neural development and plasticity (130-132). |
| 55 | P00742 | F10 | Coagulation factor X (F10) is a vitamin K-dependent serine protease which in its activated state converts prothrombin to thrombin for blood clotting (133). |
| 56 | P01133 | EGF | Epidermal Growth Factor (EGF) is a mitogenic factor that binds to the plasma membrane EGF receptor (EGFR) and stimulates cell growth and differentiation signalling pathways (134). |
| 57 | Q7Z3D4 | LYSMD3 | LysM Domain Containing 3 (LYSMD3) is a transmembrane protein expressed on the surface of airway epithelial cells that serves as a pattern recognition receptor and binds chitin and fungi to release cytokines (135). |
| 58 | Q9Y5K8 | ATP6V1D | ATPase H+ Transporting V1 Subunit D (ATP6V1D) is a part of vacuolar ATPase (V-ATPase) responsible for organelle acidification (84). |

**Supplemental Table 4. Organ System Expression Keyword Categories and NLP Associated Proteins**

| **Type** | **Proteins** | **Keywords** |
| --- | --- | --- |
| Respiratory | ABHD14B, ARG1, BDNF, BMP4, CREBZF, CXCL11, EDF1, FCN1, PDGFB | airway epithelium, airways, alveolar walls, bronchi, bronchial epithelial, bronchial glands, bronchial submucosal, bronchiolar epithelium, bronchioles, bronchus, bronchus-associated, embryonic lung, fetal lung, larynx, lung, lung endothelium, lung parenchyma, lung submucosal, lung submucosal gland acinus, lung vascular smooth muscle, lungs, nasal, nasal cavity, nasal mucosa, nasal septal epithelium, nasopharynx, pharynx, pseudostratified epithelia, pulmonary, pulmonary airways, pulmonary alveoli, respiratory epithelium, respiratory tract, respiratory tracts, ribs, sinonasal, submucosal serous, trachea |
| Urinary | ABHD14B, ATP6V1G1, BMP4, CREBZF, EDF1, EGF, PDGFB, SORT1, SOST | ascending limbs, bladder, bladder urothelium, collecting duct, collecting ducts, collecting tubule, convoluted tubule, convoluted tubule lumen, convoluted tubules, cortical collecting tubules, descending limbs, distal tubules, fetal bladder, fetal kidney, fetal kidneys, glomeruli, glomerulus, henle, kidney, kidney artery, kidney cortex, kidney distal, kidney glomeruli, kidney medulla, kidneys, mesangium, metanephric mesenchyme, nephron, nephron segments, non tumor kidney, normal kidney, perirenal adipose, proximal tubule, proximal tubules, renal proximal tubule, renal proximal tubules, skeletal muscle kidney, transitional epithelia, tubular epithelium, ureter, urinary bladder, urogenital, urothelium, vas deferens |
| Reproductive | ABHD14B, BDNF, BMP4, CASP8, CXCL11, EGF, KLK13, SIAE, SORT1 | adult testis, bartholin's, breast, breast cyst, cerebrum, cervical, cervical squamous epithelium, cervix, chorioamniotic mesenchyma, chorion, chorionic villi, colon testis, cytotrophoblast, decidua, ectocervical epithelium, embryo testis, endocervical glands, endometrial fluid, endometrial stroma, endometrium, endometrium basalis, endometrium epithelium, epididymal duct epithelia, epididymal glands, epididymis, epididymis lumen, excurrent ducts, fallopian tube, fallopian tubes, female reproductive, fetal testis, foreskin, genital, genital tract, gingival crevicular, gingival crevicular fluid, gonadal ridge, graaf follicle fluids, isthmus, mammary, mammary epithelia, mammary epithelial cell surfaces, mammary gland, mammary glands, myometrium, neoplastic prostate, nipple aspirate, nipple epidermis, outer myometrial smooth muscle, ovarian, ovaries, ovary, oviduct, penis, prostate, prostate epithelium, prostate fibromuscolar stroma, prostate gland, prostrate, reproductive, reproductive system, seminal vesicle, seminal vesicles, seminiferous tubules, syncytiotrophoblast, testes, testicles, testis, transitional epithelia, transitional epithelium, umbilical chord, umbilical cord, uterine endometrium, uterine fluid, uterine glandular epithelium, uterus, vagina, vaginal epithelium |
| Cardiovascular | ABHD14B, BDNF, CA4, CREBZF, EDF1, PDGFB, SORT1, SOST | abdominal aorta, aorta, aorta extracellular, aortic, aortic intima, aortic valves, arteries, arteriolar tree, artery, ascending aorta, atria, atrium, atrium cardiomyocytes, blood, blood vessel walls, blood vessels, capillaries, capillary endothelium, cardiac, cardiac atria, cardiac muscle, cardiac muscles, cardiovascular, clood vessels, coronary, coronary arteries, coronary artery, coronary artery smooth muscle, dermal blood vessels, ductus arteriosus, endothelia, endothelial, endothelial cell layer, endothelial venules, endothelium, fetal heart, heart, heart muscle, heart spleen, heart ventricle, heart ventricles, hearts, inter-ventricular septum, large arteries, large vessels, lateral ventricle, liver heart, mammary artery, myocardium, normal endothelium, outflow tract, periosteum, right atrium, right ventricle, skin blood vessels, small capillaries, small vessel endothelium, small vessels, stromal vascular, subendothelial layer, subendothelial layers, system vessels, thoracic aorta, umbilical cord artery, umbilical cord vascular smooth muscle, vasa vasorum, vascular, vascular capillary network, vascular endothelium, vascular smooth muscle, vascular structure, vascular structures, vascular system, vascular-rich organs, vasculature, vein, veins, ventricle, ventricles, ventricular, ventricular trabeculae, vessel wall, vessels, visceral smooth muscle |
| Musculoskeletal | ABHD14B, BDNF, CASP8, CREBZF, ENO1, NEB, PDGFB, SORT1, SOST | adult skeletal muscle, appendicular skeleton, articular, articular cartilage, articular cartilages, articular hyaline cartilage, bone, bone matrix, bone-forming sites, bone-forming surfaces, bones, calvaria, carpal bones, cartilage, cartilages, cartilaginous, cartilaginous cores, cortical plate, cranial cartilage, deep zone cartilage, dental enamel, dental papilla, dental pulp, dentin, epiphysis, fetal cartilage, fetal perichondrium, ganglia, hip articular cartilage, hypertrophic cartilage, inner periosteal region, intervertebral disk, invertebral disk, joint capsule, joint cartilage, joints, junctional epithelium, ligament, ligaments, long bone, long bones, lumbar disk, metaphyseal bone, muscle, muscle fibers, muscles, oa lesions, osseous, osteoarthritic, osteoarthritic articular cartilage, osteoarthritic synovium, perichondrium, periodontium, pulp, rheumatoid synovial, rib bone, rod-interrod enamel, sarcomeric muscle, skeletal, skeletal muscle, skeletal muscle kidney, skeletal muscle), skeletal muscles, smooth muscle, smooth muscles, spinal muscular, striated muscle, striated muscles, superficial zone, synovial, synovial fluid, synovial membranes, synovium, tarsal bones, tendon, trabecular bone, vertebrae, visceral smooth muscle |
| Endocrine | ABHD14B, BDNF, CXCL11, DKK1, EDF1, HPSE, PDGFB, SORT1 | adipose, adrenal, adrenal cortex, adrenal gland, adrenal glands, adrenal glomerulosa, adult placenta, brown adipose, ducts, endocrine, endocrine glands, endocrine secretory, fat, fetal adrenal, fetal placenta, gland, glands, hypophysis, hypothalamus, intestinal glands, lacrimal gland, lateral hypothalamus, parathyroid, parathyroid gland, phaeochromocytoma, pineal gland, pituitary, pituitary gland, placenta, placenta syncytiotrophoblasts, placenta vascular, placenta vascular endothelium, placental, placental endothelium, placental membranes, placental stem villi vessels, placental vascular, placental villi, placentas, secretory epithelia, steroidogenic glands, subcutaneous adipose, subcutaneous fat, submucosal gland, submucosal glands, term placenta, thyroid, thyroid follicles, thyroid gland, thyroid glands, visceral adipose, white adipose, white fat |
| Digestive | ABHD14B, CASP8, CLEC1B, CREBZF, CXCL11, EDF1, EGF, F10, HPSE, ITIH4, KLK13, SIAE, SORT1, SOST | adult colon epithelium, adult pancreas, adult stomach, appendix, bile ducts, bowel, brush border, buccal mucosa, cecum, colon, colon intestine, colon mucosa, colon parasympathetic ganglia, colon testis, colon-rectum muscolaris mucosae epithelium, colonic crypts, colonic epithelium, colonic lymphoid follicles, colonic mucosa, colorectal, crypts, deodenum, descending colon, digestive, digestive system epithelium, duodenum, duodenum mucosal crypts, esophagus, exocrine pancreas, exocrine pancreatic ducts, fetal colon, fetal liver, fundic epithelium, gall bladder, gallbladder, gallbladder bile, gastric, gastric antrum, gastric mucosa, gastrointestinal, gastrointestinal epithelia, gastrointestinal epithelium, gastrointestinal tract, gingival mucosa, gut, gut lamina propria, hepatic, hepatic endothelia, ileocecum, ileum, intestinal, intestinal brush border, intestinal crypts, intestinal epithelia, intestinal epithelium, intestinal tract, intestine, intestines, islets, jejunum, jejunum brush border, langerhans, large intestine, liver, liver heart, liver hepatocytes, liver pancreas, liver sinuses, liver skeletal muscle, mouth, mucous acini, non-cancerous liver, normal stomach, omental, omentum, oral cavity, oral epithelia, oral epithelium, oral mucosa, oral tongue, palatal epithelia, palatal shelf, palate, pancreas, pancreas acinar ductal epithelium, pancreas islets, pancreatic, pancreatic acini, pancreatic beta-cells, pancreatic duct, pancreatic islets, papillae, parotid, parotid gland, parotid salivary gland intralobular ducts, rectal, rectum, salivary, salivary gland, salivary glands, sinusoidal epithelium, small instestine, small intestine, small intestines, stomach, stomach wall, sublingual gland, sublingual glands, submandibular, submandibular gland, submaxillary glands, teeth, tongue, tongue mesenchyme, tooth, transverse, transverse colon, villi, villous stroma |
| Lymphatic | ABHD14B, CASP8, CXCL11, FCN1, HPSE, SORT1, SOST | splenic-rich red pulp, adenoid, adult lymph nodes, bone marrow, fetal spleen, fetal thymus, fetal tonsils, germinal center, germinal centers, hematopoietic, immune system, interfollicular regions, lymph, lymph node, lymph node-containing, lymph nodes, lymphatic, lymphatic vessels, lymphatics, lymphocytic compartment, lymphoid, lymphoid node, lymphoid organs, mantle zones, mesenteric lymph nodes, peripheral lymph nodes, peyer patches, peyer's patches, peyers's patches, red pulp, secondary lymphoid, spleen, thymic medulla, thymus, thymus epithelium, thymus medulla, tonsil, tonsils |
| Nervous System | BDNF, CA4, CASP8, DCTN1, EDF1, PDGFB, PTN, SAMD9L, SERPINI1, SORT1 | blood brain barrier, adrenal medulla, adult cns, adult nervous central system, amygdala, anterior horn, anterior perisylvian cortex-opercular gyri, auerbach plexus, axons, basal ganglia, blood-brain, brain, brain cortex, brain neocortex, brain placenta, brain regions, brain stem, brain structures, brainstem, bruch's membrane, ca1, ca3, ca3 regions, caudate nuclei, caudate nucleus, caudate region, central, central nervous, central nervous system, cerebellar nuclei, cerebellum, cerebral cortex, cerebral spinal, cerebro-spinal fluid, choriocapillaris, choroid, choroid plexus, ciliary body, ciliary border, ciliary nonpigmented epithelium, circumvallate papillae, cns, cochlea, cone photoreceptors, conjunctival epithelia, conjunctival epithelium, cornea, corneal, corneal epithelium, corpus callosum, corpus luteum, corpus region, cortex, cortical layers, cranial ganglia, dentate gyrus, dentate nucleus, diencephalon, dorsal root ganglia, dorsal root ganglion, drg, embryonic retina, extraocular smooth muscle, eye, eye anterior segment, eye lens, eyes, fetal brain, fetal brains, fetal cerebellum, fetal eye, fetal frontal lobe, fetal retinal pigment epithelium, fetal substantia nigra, frontal cortex, frontal lobe, fusiform gyrus, ganglion cell layer, germinal neuroepithelium, globus pallidus, hippocampal ca1, hippocampal dentate gyrus, hippocampal subfields, hippocampus, inner ear, inner ear structures, inner nuclear layer, inner plexiform layer, inner segments, inner uveal meshwork, insula, iris, lumbar, medulla, medulla oblongata, medulla region, midbrain structures, motor cortices, myelinated structures, neocortex, neocortical regions, nerve fiber layer, nervous, nervous system, neural, neural retina, neuron, neuronal, neutrophils thyroid gland, notochord, nucleus accumbens, occipital, occipital lobe, occipital pole, olfactory bulb, olfactory epithelium, olfactory lobe, olfactory tubercles, ophthalmic nerve, optic nerve, outer nuclear layer, outer plexiform layer, papillary sphincter, parahippocampal cortex, paraolfactory gyri, parietal lobe, parietal lobes, periaxonal myelin, peripheral nerve, peripheral nervous system, peripheral nervous systems, peripheral retina, periventricular, photoreceptor outer, pigmented epithelium, pns neuroectoderm, pons, posterior perisylvian, postrema, postsynaptic structures, prefrontal cortex, putamen, pyramidal cell layer, retina, retina pigment epithelium, retinal, retinal cone photoreceptors, retinal pericytes, retinal pigment epithelia, retinal pigment epithelium, retinal rod, rod, rod photoreceptors, rolandic area, rostral segment, sclera, spinal chord, spinal cord, spinal cordon, stratum pyramidale, stria vascularis, striate, striated tracts, striatum, subiculum, substantia nigra, subthalamic nucleus, subventricular zones, sustantia nigra, sympathetic, telencephalon, temporal cortex, temporal gyrus, temporal lobe, temporal lobes, thalamus, ventral striatum, vertebrae, vestibular system, vestibule |
| Integumentary | CCL5, CXCL11, HPSE | anagen follicles, basal epidermal layer, basal layer, basal skin layer, body wall, club hair, dermal papilla, dermis, eccrine sweat, eccrine sweat glands, epidermal, epidermal-dermal junction, epidermis, epithelial layer, exocrine, exocrine secretions, fetal follicles, fetal skin, follicular, follicular fluid, hair fibers, hair follicle, hair follicles, huxley's layers, inner endocuticle, inner root sheath, interfollicular epidermis, keratinocyte layers, lesional psoriatic skin, nail bed epithelium, nail matrix, outer root sheath, palmoplantar epidermis, root sheath, scalp, scalp follicles, scalp skin, scar lesional skin, sebaceous gland, skin, skin epidermis, spinosum, stratum corneum, stratum granulosum, stratum spinosum, subcutaneous, suprabasal layers, suprabasilar layers, sweat, sweat ducts, sweat gland, sweat gland ducts, sweat glands, upper spinous layers |

**Supplemental Table 5. Cell Type Expression Keyword Categories and NLP Associated Proteins**

| **Type** | **Proteins** | **Keywords** |
| --- | --- | --- |
| Leukocyte NYD | ABHD14B, ARG1, CLEC1B, FCN1, HLA-DRA, HPSE, SERPINA1 | ag-presenting, apcs, blood leukocytes, blood mononuclear, bone, bone marrow, bone marrow-derived, bone marrow-derived mesenchymal stem, bone trabecular, bone-derived, cortical thymocytes, flattened bone-lining, immune, immunoblasts, immunocyte lines, interstitial leukocytes, leukocyte, leukocyte lines, leukocytes, mast, mononuclear, mononuclear leukocytes, myeloblast, normal mast, pbmc, pbmcs, peripheral blood leukocyte, peripheral blood leukocytes, peripheral blood mononuclear, peripheral blood mononuclear leukocytes, peripheral leukocyte, peripheral leukocytes, peripheral mononuclear, phagocytes, phagocytic, plasma, pmns, polymorphonuclear leukocytes, promyelocyte stage, promyelocytes, promyelocytic, submucosal leukocytes, thymocytes, urothelial |
| Lymphocyte | ARG1, CCL5, GLI2, HLA-DRA, LTA4H | t-differentiated hl-60, alpha-beta t, b, b lineage, b-, b- lineage, b- lines, b-1, b-lymphocyte, b-lymphocytes, b-lymphoid lines, b-lymphomas, blood lymphocytes, ca4, cytolytic, cytotoxic t lymphocytes, cytotoxic t-lymphocytes, decidual nk, effector, epstein-barr virus-transformed lymphoblastoid lines, fetal nk-, gamma delta t, gamma-delta t, gamma-delta t-, germinal center, germinal center b-, germinal centers, group2 innate lymphoid, helper t-, hut 78, ilc2s, intraepithelial lymphocytes, intratumoral nk, jurkat, jurkat lines, jurkat t- leukemia, jurkat t- line, large lymphocytes, lymphoblasts, lymphocyte, lymphocytes, lymphocytic lines, lymphocytic lineage, lymphoid lines, lymphoid organs, marginal zone b-, mature b, melanoma-specific cytotoxic t clones, memory b-, memory gamma-delta t, memory t-, memory th17, molt-4, molt-4 lines, naive t, natural killer, natural killer (nk), neoplastic b- and t- lines, nk, nk subsets, nk- line, nkt, normal germinal center (gc) b-, pbl, peripheral blood lymphocytes, peripheral blood memory t-, peripheral blood t-, peripheral blood t-lymphocytes, peripheral lymphocytes, peripheral memory, peripheral t-, plasma b-, pre b-, pre t-, pre-b-, pro-b precursors, raji b-lymphoblasts, reed-sternberg (hrs), sup-t1, t, t populations, t lymphocytes, t-, t- clones, t- leukemia lines molt-4, t- lineage, t- lines, t- lines harris, t- lymphoid lines, t- subsets, t-helper, t-helper 2, t-lymphoblasts, t-lymphocytes, th0, th1, thymus-derived t-, tonsillar germinal center centrocytes, transitional b, treg, treg) |
| Macrophage | ARG1, CCL5, FCN1, HLA-DRA | alveolar macrophages, bone marrow macrophages, cd68, cortical macrophages, decidual macrophages, epidermal langerhans, epidermoid, hofbauer, kg-1, kupffer, langerhans, langerhans', liver kupffer, liver kuppfer, lung alveolar macrophages, m1 macrophages, macrophage, macrophage line, macrophage lines u-937, macrophage progenitor, macrophage-like, macrophages, meningeal macrophages, monocyte-derivedmacrophages, non sec-, perivascular macrophages, placental macrophages, red pulp macrophages, spleen macrophage, tissue macrophages |
| Platelet | CLEC1B, LTA4H, VAMP8 | eosinophil platelets, megakaryoblastic, megakaryocytes, megakaryocytic, platelet, platelets, thrombocytes |
| Granulocyte | ARG1, FCN1, LTA4H | basophil, basophils, bone marrow neutrophils, eosinophils, granular, granule, granulocyte, granulocytes, granulocytic, inflammatory, neutrophil, neutrophil lineage, neutrophils, peripheral blood granulocytes, peripheral blood neutrophils, polynuclear neutrophils |
| Monocyte | FCN1, LTA4H | cd11b monocytes, mono-mac-6, monocyte, monocyte-related, monocytes, monocytic, myelomonocytic, myelomonocytic lineage, peripheral blood monocytes, promonocytic, thp-1, thp-1 monocytes |
| Cancer | BMP4, MRPL58 | epidermoid carcinoma, acute myelocytic leukemia, acute myelogenous leukemia), acute myeloid leukemia, all leukemia/lymphoma lines, bladder cancer, bladder carcinoma, breast cancer lines, breast cancer lines mcf-7, breast cancer lines mda-mb-231, breast cancer lines, breast carcinoma lines, cancer, cancer lines, carcinoma, carcinoma lines, choriocarcinoma, choriocarcinoma cancer lines, colon adenocarcinoma line t84, colon adenocarcinoma lines, colon cancer lines, colorectal adenocarcinoma line, colorectal cancer, colorectal cancer lines, erythroleukemia, erythroleukemia line k-562, fa6, fibrosarcoma, gastric, gastric cancer lines, glioblastoma, glioblastoma lines, glioblastomas, hairy leukemia, hbl-100 breast carcinoma, hel, hematopoietic tumor lines, hepatoular carcinoma, hl-60, hodgkin, hpaf, hs 294t melanoma, ht29-d4 colon carcinoma, imim-pc2, intratumoral nk, k-562, k-562 erythroleukemia, kidney tumor, leiomyomal, leukemia lines, leukemia u-937, leukemia u-937 line, leukemic, leukemic lines, lung carcinoma lines, lung tumor lines, lymphoma, lymphoma lines, malignant, malignant hodgkin lymphoma, malignant melanoma, malignant melanoma lines, mammary carcinoma lines, mcf-7 breast carcinoma, mda-mb-175, mda-mb-435, melanoma, melanoma lines, metastasizing melanoma lines, myelogenous leukemia line kg-1, myelogenous leukemic lines, myeloid leukemia lines, nb4, neoplastic lines, neuro-epithelioma, neuroblastoma, non invasive breast carcinoma lines, non-glial-derived nervous system tumor lines, non-hodgkin lymphoma lines, noneuroblastoma, nurse-like, pancreatic cancer lines, pancreatic carcinoma lines, panctu-ii, paraneoplastic tumor, pc-3, promyelocytic leukemia line hl-60, prostate cancer, prostate cancer lines, prostatic adenocarcinoma lines, retinoblastoma lines, several cancer lines, sk-ov-3 (ovary adenocarcinoma), snu-c2b colon carcinoma, sw48, sw480, sw480 colon carcinoma, sw480 colorectal cancer line, testicular tumor, tumor, tumor lines, tumor endothelial, tumor invasive tumors, tumor-derived lines, tumoral, tumors lines, u-937 histiocytic lymphoma lines |
| Osteoblast | PTN, SOST | bone osteoblasts, giant osteoclast-like, osteoblast, osteoblast line mg-63, osteoblast line saos-2, osteoblast-like, osteoblasts, osteoclasts, osteocytes, osteogenic, osteosarcoma lines, primary ossification center-associated, subchondral bone osteoblasts |
| Neuron | ENO1, SERPINI1 | basket, brain neurons, ca2, central neurons, cerebellar purkinje, cerebral cortex, cortical neurons, dentate gyrus granule neurons, dopaminergic neurons, dorsal root ganglia neurons, dorsal root ganglion, gabaergic neurons, granule neurons, gray matter neurons, hippocampal pyramidal neurons, hippocampus pyramidal, hippocampus pyramidal neurons, lines, neocortical neurons, neural, neural crest, neural progenitor, neural stem, neuroendocrine, neuroendocrine epithelium, neuron, neuronal, neurons, olfactory receptor neurons, peripheral, peripheral neurons, pontine nuclei, purkinje, purkinje neurons, pyramidal, pyramidal neurons, retinal ganglion, schwann, schwann culture, spinal cord neurons, stellate, striatal neurons |
| Skin | CXCL11 | basal keratinocytes, cornified, epidermal basal layer keratinocytes, epidermal keratinocytes, keratinocyte, keratinocytes, megakaryocytic lines, melanocyte, melanocytes, pigment, skin keratinocytes, suprabasal keratinocytes |
| Myeloid | CLEC1B | chronic myelogenous, early myeloid lines, monocytic/myeloid lineage, mucosal myeloid, myeloid, myeloid lines, myeloid lineage, myeloid lineages, myeloid progenitor, myeloid-derived suppressor, myeloids, myeloma, myeloma line u266b1, myeloma line u266r |
| Glial | CXCL11 | astrocytes, astrocytoma, glia, glial, glioma, glioma lines, glioma tissue, liver astrocytes, microglia, microglial, neuro-glial, olgs, oligodendrocytes, oligodendroglia, perivascular astrocytes |
| Adipocyte | C3 | adipocyte, adipocytes |
| Other Blood | LTA4H | blood, blood lines, cord blood, peripheral blood, red blood, reticulocytes |
| Fibroblast | LTA4H | cerebral pericytes, dermal fibroblasts, fetal fibroblasts, fibroblast, fibroblast lines, fibroblast-like synoviocytes, fibroblastic, fibroblasts, foreskin fibroblast, foreskin fibroblasts, gingival fibroblasts, myofibroblasts, pulmonary fibroblasts, skin fibroblasts, stromal fibroblast, stromal fibroblasts, synovial, synovial fibroblasts, synovial fluid |
| Epithelial | HLA-DRA | airway epithelial, alveolar epithelial, alveolar type 2, alveolar type ii, antral epithelial, atypical epithelial, breast epithelial, breast epithelial line mcf-10a, breast epithelial lines, bronchial, bronchial epithelial, choroid plexus epithelial, ciliary body epithelial, ciliated, colonic, colonic epithelial, columnar epithelial, corneal epithelial, embryonic epithelial, epithelial, epithelial lines, eue, gastric epithelial, gastrointestinal epithelial, hek293, hela, helas3, hep-g2, ht-29 colonic epithelial, intestinal epithelial, intraepithelial cd8-positive t, intraepithelial lymphocytes, kidney epithelial, kidney proximal tubular epithelial, luminal epithelial, lung alveolar type 2, lung epithelial, mammary epithelial, mcf-7, mesothelial, myoepithelial, myoepithelium, nasal, nasal epithelial, nonciliated, paneth, parietal epithelial, pharyngeal epithelial, prostate gland epithelial, renal proximal tubule epithelial, retina pigment epithelial, retinal pigment epithelial, secretory epithelial, small intestinal epithelial, surface epithelial, t-47d, thymic, thymic epithelial, thyrocytes, tracheal surface epithelial, tubular epithelial, type ii alveolar, unpolarized epithelial, vascular epithelial, zr-75-1 |
| Dendritic | HLA-DRA | cutaneous dendritic, dc, dendritic, follicular dendritic, ikdcs, imddc, immature dendritic, interdigitating reticulum, interferon-producing killer dendritic, mddc, monocyte-derived, monocyte-derived dendritic, myeloid dendritic, pdc, pdcs, peripheral blood plasmacytoid dendritic, plasmacytoid, plasmacytoid dendritic, plasmacytoids, thymic dendritic, tolerogenic dcs, tonsil dc, tonsil interdigitating dendritic, various dendritic |
| Endothelial | HPSE | angioblasts, aorta endothelial, aortic endothelial, arterial endothelial, artery endothelial, blood brain barrier endothelial, cervical epithelium, umbilical vein endothelial, endiothelial, endothelial, endothelial venules, human umbilical vein endothelial, huvecs, liver sinusoidal endothelial, lung endothelial, lymph vessel endothelial, microvascular capillary endothelial, microvessels endothelial, placenta, umbilical vein endothelial, renal glomeruli endothelial, reticuloendothelial, sinusoidal endothelial, tumor endothelial, umbilical veil endothelial, umbilical vein endothelial, vascular endothelial |
| Chondrocyte | | articular chondrocytes, chondrocyte, chondrocyte-like, chondrocytes, fetal chondrocyte |
| Dental |  | ameloblast, cementoblast, odontoblast, ondotoblasts |
| Endocrine |  | enterocyte-like, enterocytes, enteroendocrine, enteroendocrine l, ileal absorptive enterocytes |
| Erythrocyte | | erythroblasts, erythrocytes, erythroid, fetal erythrocytes |
| Eye |  | amacrine, cone, photoreceptors, rod photoreceptor |
| Hematopoietic | | hematopoetic, hematopoietic, hematopoietic lines, hematopoietic lineage, hematopoietic precursors, hematopoietic progenitor, hematopoietic stem, hemopoietic |
| Non-Hematopoietic | | non-hematopoietic |
| Kidney |  | bowman's capsule, distal tubular, embryonal kidney, embryonic kidney, glomerular epithelium, glomerular mesangial, hsc, interstitial, kidney distal tubular, mesangial, podocyte, podocytes, proximal tubule, renal, renal lines, renal proximal tubular, tubular |
| Liver |  | crypt, hepatic, hepatic parenchymal, hepatic stellate, hepatocytes, hepatoma, hepatoma lines, liver hepatocytes |
| Mucous |  | bronchial goblet, ciliated bronchiolar, goblet, intestinal mucosa, mucous, mucus-secreting, nasal goblet secretory, upper gastric mucosal |
| Muscle |  | airway smooth muscle, aortic smooth muscle, arterial smooth muscle, artery smooth muscle, cardiac, cardiac myocytes, cardiomyocytes, coronary muscle, myoblasts, myocytes, myotubes, perivascular, placental vascular smooth muscle, pulmonary artery smooth muscle, skeletal muscle, smooth, smooth muscle, umbilical vein smooth muscle, vascular, vascular smooth, vascular smooth muscle, vascular wall, vsmc |
| Pancreatic |  | beta-, ductile, islet, pancreas islet beta, pancreatic, pancreatic lines, pancreatic acinar, pancreatic beta, pancreatic islet |
| Reproductive | | cumulus, cyto- and syncytiotrophoblastic, cytotrophoblasts, extravillous trophoblast, germ, gonocytes, granulosa, interstitial leydig, leydig, luteinized granulosa, migratory primordial germ, myometrial, oocytes, ovarian granulosa, ovary, postnatal leydig, prostate, secretory endometrial, sertoli, spermatocytes, spermatogenic, spermatogonia, spermatogonias, spermatozoa, syncytiotrophoblast, syncytiotrophoblasts, trophoblast, trophoblasts, x |
| Secretory |  | chief, chromaffin, clara, enterochromaffin, fundic, gastric parietal, secretory, serous-like, weibel-palade bodies, zymogen-producing |
| Spleen |  | spleen, splenic marginal zone |
| Stem |  | cambial, cml) stem, embryonic stem, esc, mesenchymal, mesenchymal stem, stem |
| Stromal |  | bone marrow stromal, endometrial stromal, stromal, stromal type |

**References**

1. Chang A*, et al.* (2002) Human kallikrein gene 13 (KLK13) expression by quantitative RT–PCR: an independent indicator of favourable prognosis in breast cancer. *British Journal of Cancer* **86:** 1457-1464.

2. Lin Q*, et al.* (2017) Downregulation of KLK13 promotes the invasiveness and metastasis of oesophageal squamous cell carcinoma. *Biomedicine & Pharmacotherapy* **96:** 1008-1015.

3. Tokas T*, et al.* (2017) Downregulated KLK13 expression in bladder cancer highlights tumor aggressiveness and unfavorable patients’ prognosis. *Journal of Cancer Research and Clinical Oncology* **143:** 521-532.

4. Milewska A*, et al.* (2020) Kallikrein 13 serves as a priming protease during infection by the human coronavirus HKU1. *Sci Signal* **13**.

5. Haeggström JZ, Kull F, Rudberg PC, Tholander F, Thunnissen MMGM. (2002) Leukotriene A4 hydrolase. *Prostaglandins & Other Lipid Mediators* **68-69:** 495-510.

6. Penning TD. (2001) Inhibitors of leukotriene A4 (LTA4) hydrolase as potential anti-inflammatory agents. *Curr Pharm Des* **7:** 163-179.

7. Vo TTL, Jang WJ, Jeong CH. (2018) Leukotriene A4 hydrolase: an emerging target of natural products for cancer chemoprevention and chemotherapy. *Ann N Y Acad Sci* **1431:** 3-13.

8. Ning Z, Liu K, Xiong H. (2021) Roles of BTLA in Immunity and Immune Disorders. *Frontiers in Immunology* **12**.

9. Al-Mterin MA, Alsalman A, Elkord E. (2022) Inhibitory Immune Checkpoint Receptors and Ligands as Prognostic Biomarkers in COVID-19 Patients. *Front Immunol* **13:** 870283.

10. Herrmann M*, et al.* (2020) Analysis of Co-inhibitory Receptor Expression in COVID-19 Infection Compared to Acute Plasmodium falciparum Malaria: LAG-3 and TIM-3 Correlate With T Cell Activation and Course of Disease. *Front Immunol* **11:** 1870.

11. Schultheiß C*, et al.* (2020) Next-Generation Sequencing of T and B Cell Receptor Repertoires from COVID-19 Patients Showed Signatures Associated with Severity of Disease. *Immunity* **53:** 442-455.e444.

12. Shubin NJ, Monaghan SF, Heffernan DS, Chung C-S, Ayala A. (2013) B and T lymphocyte attenuator expression on CD4+ T-cells associates with sepsis and subsequent infections in ICU patients. *Critical Care* **17:** R276.

13. Wang Z, Wesche H, Stevens T, Walker N, Yeh W-C. (2009) IRAK-4 inhibitors for inflammation. *Current topics in medicinal chemistry* **9:** 724-737.

14. Picard C, Casanova J-L, Puel A. (2011) Infectious Diseases in Patients with IRAK-4, MyD88, NEMO, or IκBα Deficiency. *Clinical Microbiology Reviews* **24:** 490-497.

15. Umar S*, et al.* (2022) Inhibition of IRAK4 dysregulates SARS-CoV-2 spike protein-induced macrophage inflammatory and glycolytic reprogramming. *Cell Mol Life Sci* **79:** 301.

16. Yang CA, Huang YL, Chiang BL. (2022) Innate immune response analysis in COVID-19 and kawasaki disease reveals MIS-C predictors. *J Formos Med Assoc* **121:** 623-632.

17. Fisher AB. (2017) Peroxiredoxin 6 in the repair of peroxidized cell membranes and cell signaling. *Arch Biochem Biophys* **617:** 68-83.

18. Hu X, Lu E, Pan C, Xu Y, Zhu X. (2020) Overexpression and biological function of PRDX6 in human cervical cancer. *J Cancer* **11:** 2390-2400.

19. Huang WS*, et al.* (2018) Expression of PRDX6 Correlates with Migration and Invasiveness of Colorectal Cancer Cells. *Cellular Physiology and Biochemistry* **51:** 2616-2630.

20. López-Mateo I, Villaronga M, Llanos S, Belandia B. (2012) The transcription factor CREBZF is a novel positive regulator of p53. *Cell Cycle* **11:** 3887-3895.

21. Hu Z*, et al.* (2020) CREBZF as a Key Regulator of STAT3 Pathway in the Control of Liver Regeneration in Mice. *Hepatology* **71:** 1421-1436.

22. Hu Z*, et al.* (2019) 265-OR: Myeloid CREBZF Couples Inflammatory Signals to Systemic Insulin Resistance by Regulating Immune Response of the Adipose Tissue. *Diabetes* **68:** 265-OR.

23. Mao B*, et al.* (2001) LDL-receptor-related protein 6 is a receptor for Dickkopf proteins. *Nature* **411:** 321-325.

24. Kamiya N. (2012) The role of BMPs in bone anabolism and their potential targets SOST and DKK1. *Current molecular pharmacology* **5:** 153-163.

25. Sahu A, Lambris JD. (2001) Structure and biology of complement protein C3, a connecting link between innate and acquired immunity. *Immunological Reviews* **180:** 35-48.

26. Russell AJ*, et al.* (2021) SAMD9L autoinflammatory or ataxia pancytopenia disease mutations activate cell-autonomous translational repression. *Proceedings of the National Academy of Sciences* **118:** e2110190118.

27. Kim JJ*, et al.* (2020) Identification of SAMD9L as a susceptibility locus for intravenous immunoglobulin resistance in Kawasaki disease by genome-wide association analysis. *Pharmacogenomics J* **20:** 80-86.

28. Brindle NPJ, Saharinen P, Alitalo K. (2006) Signaling and Functions of Angiopoietin-1 in Vascular Protection. *Circulation Research* **98:** 1014-1023.

29. Thurston G. (2002) Complementary actions of VEGF and Angiopoietin-1 on blood vessel growth and leakage*. *Journal of Anatomy* **200:** 575-580.

30. Davis S*, et al.* (1996) Isolation of Angiopoietin-1, a Ligand for the TIE2 Receptor, by Secretion-Trap Expression Cloning. *Cell* **87:** 1161-1169.

31. Cole KE*, et al.* (1998) Interferon-inducible T cell alpha chemoattractant (I-TAC): a novel non-ELR CXC chemokine with potent activity on activated T cells through selective high affinity binding to CXCR3. *J Exp Med* **187:** 2009-2021.

32. Ehlert JE, Gerdes J, Flad HD, Brandt E. (1998) Novel C-terminally truncated isoforms of the CXC chemokine beta-thromboglobulin and their impact on neutrophil functions. *J Immunol* **161:** 4975-4982.

33. Wismans LV*, et al.* (2023) Increase of mast cells in COVID-19 pneumonia may contribute to pulmonary fibrosis and thrombosis. *Histopathology* **82:** 407-419.

34. Yatim N*, et al.* (2021) Platelet activation in critically ill COVID-19 patients. *Ann Intensive Care* **11:** 113.

35. ten Dijke P*, et al.* (1994) Identification of type I receptors for osteogenic protein-1 and bone morphogenetic protein-4. *J Biol Chem* **269:** 16985-16988.

36. Rezzola S*, et al.* (2019) VEGFR2 activation mediates the pro-angiogenic activity of BMP4. *Angiogenesis* **22:** 521-533.

37. Semënov M, Tamai K, He X. (2005) SOST is a ligand for LRP5/LRP6 and a Wnt signaling inhibitor. *J Biol Chem* **280:** 26770-26775.

38. Delgado-Calle J, Sato AY, Bellido T. (2017) Role and mechanism of action of sclerostin in bone. *Bone* **96:** 29-37.

39. Brunkow ME*, et al.* (2001) Bone Dysplasia Sclerosteosis Results from Loss of the <em>SOST</em> Gene Product, a Novel Cystine Knot&#x2013;Containing Protein. *The American Journal of Human Genetics* **68:** 577-589.

40. Balemans W*, et al.* (2002) Identification of a 52 kb deletion downstream of the <em>SOST</em> gene in patients with van Buchem disease. *Journal of Medical Genetics* **39:** 91-97.

41. Balemans W*, et al.* (2001) Increased bone density in sclerosteosis is due to the deficiency of a novel secreted protein (SOST). *Human Molecular Genetics* **10:** 537-544.

42. Fredriksson L, Li H, Eriksson U. (2004) The PDGF family: four gene products form five dimeric isoforms. *Cytokine Growth Factor Rev* **15:** 197-204.

43. Risau W*, et al.* (1992) Platelet-Derived Growth Factor is Angiogenic In Vivo. *Growth Factors* **7:** 261-266.

44. Kazmierski ST*, et al.* (2003) The Complete Mouse Nebulin Gene Sequence and the Identification of Cardiac Nebulin. *Journal of Molecular Biology* **328:** 835-846.

45. Chu M, Gregorio CC, Pappas CT. (2016) Nebulin, a multi-functional giant. *Journal of Experimental Biology* **219:** 146-152.

46. Diao J*, et al.* (2015) ATG14 promotes membrane tethering and fusion of autophagosomes to endolysosomes. *Nature* **520:** 563-566.

47. Polgár J, Chung SH, Reed GL. (2002) Vesicle-associated membrane protein 3 (VAMP-3) and VAMP-8 are present in human platelets and are required for granule secretion. *Blood* **100:** 1081-1083.

48. Wong SH*, et al.* (1998) Endobrevin, a novel synaptobrevin/VAMP-like protein preferentially associated with the early endosome. *Mol Biol Cell* **9:** 1549-1563.

49. Cariappa A*, et al.* (2009) B cell antigen receptor signal strength and peripheral B cell development are regulated by a 9-O-acetyl sialic acid esterase. *Journal of Experimental Medicine* **206:** 125-138.

50. Surolia I*, et al.* (2010) Functionally defective germline variants of sialic acid acetylesterase in autoimmunity. *Nature* **466:** 243-247.

51. Schwegmann-Weßels C, Herrler G. (2006) Sialic acids as receptor determinants for coronaviruses. *Glycoconjugate Journal* **23:** 51-58.

52. Pillai S, Cariappa A, Pirnie SP. (2009) Esterases and autoimmunity: the sialic acid acetylesterase pathway and the regulation of peripheral B cell tolerance. *Trends in Immunology* **30:** 488-493.

53. Gettins PG. (2002) Serpin structure, mechanism, and function. *Chem Rev* **102:** 4751-4804.

54. Greene CM*, et al.* (2016) α1-Antitrypsin deficiency. *Nat Rev Dis Primers* **2:** 16051.

55. Ito K, Nakamura H, Watanabe Y. (2011) Protogenin mediates cell adhesion for ingression and re-epithelialization of paraxial mesodermal cells. *Dev Biol* **351:** 13-24.

56. Vesque C, Anselme I, Couvé E, Charnay P, Schneider-Maunoury S. (2006) Cloning of vertebrate Protogenin (Prtg) and comparative expression analysis during axis elongation. *Dev Dyn* **235:** 2836-2844.

57. Richter R*, et al.* (2010) A functional peptidyl-tRNA hydrolase, ICT1, has been recruited into the human mitochondrial ribosome. *Embo j* **29:** 1116-1125.

58. Wang F, Zhang D, Zhang D, Li P, Gao Y. (2021) Mitochondrial Protein Translation: Emerging Roles and Clinical Significance in Disease. *Frontiers in Cell and Developmental Biology* **9**.

59. Soehnlein O, Lindbom L. (2009) Neutrophil-derived azurocidin alarms the immune system. *Journal of Leukocyte Biology* **85:** 344-351.

60. Edens HA, Parkos CA. (2003) Neutrophil transendothelial migration and alteration in vascular permeability: focus on neutrophil-derived azurocidin. *Current Opinion in Hematology* **10**.

61. Tummers B, Green DR. (2017) Caspase‐8: regulating life and death. *Immunological Reviews* **277:** 76-89.

62. Frisch SM. (2008) Caspase-8: Fly or Die. *Cancer Research* **68:** 4491-4493.

63. Maelfait J, Beyaert R. (2008) Non-apoptotic functions of caspase-8. *Biochemical Pharmacology* **76:** 1365-1373.

64. Matern BM, Olieslagers TI, Voorter CEM, Groeneweg M, Tilanus MGJ. (2020) Insights into the polymorphism in HLA-DRA and its evolutionary relationship with HLA haplotypes. *HLA* **95:** 117-127.

65. Aratani Y. (2018) Myeloperoxidase: Its role for host defense, inflammation, and neutrophil function. *Archives of Biochemistry and Biophysics* **640:** 47-52.

66. Klebanoff SJ, Kettle AJ, Rosen H, Winterbourn CC, Nauseef WM. (2013) Myeloperoxidase: a front-line defender against phagocytosed microorganisms. *Journal of Leukocyte Biology* **93:** 185-198.

67. Zeng Z, Lan T, Wei Y, Wei X. (2022) CCL5/CCR5 axis in human diseases and related treatments. *Genes Dis* **9:** 12-27.

68. Wang X. (2020) Chapter Three - Pleiotrophin: Activity and mechanism. In: *Advances in Clinical Chemistry.* Makowski GS (ed.) Elsevier, pp. 51-89.

69. Imai S*, et al.* (2009) Osteocyte-derived HB-GAM (pleiotrophin) is associated with bone formation and mechanical loading. *Bone* **44:** 785-794.

70. Perez-Pinera P, Berenson JR, Deuel TF. (2008) Pleiotrophin, a multifunctional angiogenic factor: mechanisms and pathways in normal and pathological angiogenesis. *Current Opinion in Hematology* **15**.

71. Rajendran A, Vaidya K, Mendoza J, Bridwell-Rabb J, Kamat SS. (2020) Functional Annotation of ABHD14B, an Orphan Serine Hydrolase Enzyme. *Biochemistry* **59:** 183-196.

72. Rajendran A, Soory A, Khandelwal N, Ratnaparkhi G, Kamat SS. (2022) A multi-omics analysis reveals that the lysine deacetylase ABHD14B influences glucose metabolism in mammals. *J Biol Chem* **298:** 102128.

73. Iyer R*, et al.* (1998) The human arginases and arginase deficiency. *Journal of inherited metabolic disease* **21:** 86-100.

74. Sin YY, Baron G, Schulze A, Funk CD. (2015) Arginase-1 deficiency. *Journal of Molecular Medicine* **93:** 1287-1296.

75. Munder M*, et al.* (2005) Arginase I is constitutively expressed in human granulocytes and participates in fungicidal activity. *Blood* **105:** 2549-2556.

76. Neun R, Richter MF, Staeheli P, Schwemmle M. (1996) GTPase properties of the interferon-induced human guanylate-binding protein 2. *FEBS Lett* **390:** 69-72.

77. Braun E*, et al.* (2019) Guanylate-Binding Proteins 2 and 5 Exert Broad Antiviral Activity by Inhibiting Furin-Mediated Processing of Viral Envelope Proteins. *Cell Rep* **27:** 2092-2104.e2010.

78. Gill SR*, et al.* (1991) Dynactin, a conserved, ubiquitously expressed component of an activator of vesicle motility mediated by cytoplasmic dynein. *The Journal of cell biology* **115:** 1639-1650.

79. Ayloo S*, et al.* (2014) Dynactin functions as both a dynamic tether and brake during dynein-driven motility. *Nat Commun* **5:** 4807.

80. Farrer MJ*, et al.* (2009) DCTN1 mutations in Perry syndrome. *Nature Genetics* **41:** 163-165.

81. Münch C*, et al.* (2004) Point mutations of the p150 subunit of <em>dynactin</em> (<em>DCTN1</em>) gene in ALS. *Neurology* **63:** 724-726.

82. Münch C*, et al.* (2007) The p150 subunit of dynactin (DCTN1) gene in multiple sclerosis. *Acta Neurologica Scandinavica* **116:** 231-234.

83. Andrae J, Gallini R, Betsholtz C. (2008) Role of platelet-derived growth factors in physiology and medicine. *Genes Dev* **22:** 1276-1312.

84. Wang L, Wu D, Robinson CV, Wu H, Fu TM. (2020) Structures of a Complete Human V-ATPase Reveal Mechanisms of Its Assembly. *Mol Cell* **80:** 501-511.e503.

85. Koebernick K, Pieler T. (2002) Gli-type zinc finger proteins as bipotential transducers of Hedgehog signaling. *Differentiation* **70:** 69-76.

86. Didiasova M, Schaefer L, Wygrecka M. (2019) When Place Matters: Shuttling of Enolase-1 Across Cellular Compartments. *Frontiers in Cell and Developmental Biology* **7**.

87. Gemta LF*, et al.* (2019) Impaired enolase 1 glycolytic activity restrains effector functions of tumor-infiltrating CD8+ T cells. *Science Immunology* **4:** eaap9520.

88. Motamed K. (1999) SPARC (osteonectin/BM-40). *The International Journal of Biochemistry & Cell Biology* **31:** 1363-1366.

89. Rosset EM, Bradshaw AD. (2016) SPARC/osteonectin in mineralized tissue. *Matrix Biology* **52-54:** 78-87.

90. Way G, Morrice N, Smythe C, O'Sullivan AJ. (2002) Purification and Identification of Secernin, a Novel Cytosolic Protein that Regulates Exocytosis in Mast Cells. *Molecular Biology of the Cell* **13:** 3344-3354.

91. Tezuka Y*, et al.* (2018) Involvement of secernin1 in mouse development. In: *Proceedings for Annual Meeting of The Japanese Pharmacological Society WCP2018 (The 18th World Congress of Basic and Clinical Pharmacology).* Japanese Pharmacological Society, pp. PO1-13-21.

92. Miyoshi N*, et al.* (2010) SCRN1 is a novel marker for prognosis in colorectal cancer. *Journal of surgical oncology* **101:** 156-159.

93. Colonna M, Samaridis J, Angman L. (2000) Molecular characterization of two novel C-type lectin-like receptors, one of which is selectively expressed in human dendritic cells. *Eur J Immunol* **30:** 697-704.

94. Suzuki-Inoue K*, et al.* (2006) A novel Syk-dependent mechanism of platelet activation by the C-type lectin receptor CLEC-2. *Blood* **107:** 542-549.

95. Conlon DM. (2019) Role of sortilin in lipid metabolism. *Curr Opin Lipidol* **30:** 198-204.

96. Mitok KA, Keller MP, Attie AD. (2022) Sorting through the extensive and confusing roles of sortilin in metabolic disease. *J Lipid Res* **63:** 100243.

97. Rock KL*, et al.* (1994) Inhibitors of the proteasome block the degradation of most cell proteins and the generation of peptides presented on MHC class I molecules. *Cell* **78:** 761-771.

98. Domachowske JB, Dyer KD, Adams AG, Leto TL, Rosenberg HF. (1998) Eosinophil cationic protein/RNase 3 is another RNase A-family ribonuclease with direct antiviral activity. *Nucleic Acids Research* **26:** 3358-3363.

99. Pulido D, Torrent M, Andreu D, Nogués MV, Boix E. (2013) Two Human Host Defense Ribonucleases against Mycobacteria, the Eosinophil Cationic Protein (RNase 3) and RNase 7. *Antimicrobial Agents and Chemotherapy* **57:** 3797-3805.

100. Vreys V, David G. (2007) Mammalian heparanase: what is the message? *Journal of Cellular and Molecular Medicine* **11:** 427-452.

101. Fux L, Ilan N, Sanderson RD, Vlodavsky I. (2009) Heparanase: busy at the cell surface. *Trends in Biochemical Sciences* **34:** 511-519.

102. Melkonian KA, Maier KC, Godfrey JE, Rodgers M, Schroer TA. (2007) Mechanism of Dynamitin-mediated Disruption of Dynactin *. *Journal of Biological Chemistry* **282:** 19355-19364.

103. Burkhardt JK, Echeverri CJ, Nilsson T, Vallee RB. (1997) Overexpression of the dynamitin (p50) subunit of the dynactin complex disrupts dynein-dependent maintenance of membrane organelle distribution. *J Cell Biol* **139:** 469-484.

104. Anderl S, König M, Attarbaschi A, Strehl S. (2015) PAX5-KIAA1549L: a novel fusion gene in a case of pediatric B-cell precursor acute lymphoblastic leukemia. *Molecular Cytogenetics* **8:** 48.

105. Topolska-Woś AM, Chazin WJ, Filipek A. (2016) CacyBP/SIP — Structure and variety of functions. *Biochimica et Biophysica Acta (BBA) - General Subjects* **1860:** 79-85.

106. Lord MS, Melrose J, Day AJ, Whitelock JM. (2020) The Inter-α-Trypsin Inhibitor Family: Versatile Molecules in Biology and Pathology. *J Histochem Cytochem* **68:** 907-927.

107. Zhao X, Guo Y, Li L, Li Y. (2023) Longitudinal change of serum inter-alpha-trypsin inhibitor heavy chain H4, and its correlation with inflammation, multiorgan injury, and death risk in sepsis. *J Clin Lab Anal* **37:** e24834.

108. Woelfle J, Rotwein P. (2004) In vivo regulation of growth hormone-stimulated gene transcription by STAT5b. *American Journal of Physiology-Endocrinology and Metabolism* **286:** E393-E401.

109. Ferbeyre G, Moriggl R. (2011) The role of Stat5 transcription factors as tumor suppressors or oncogenes. *Biochimica et Biophysica Acta (BBA) - Reviews on Cancer* **1815:** 104-114.

110. Kaltenecker D*, et al.* (2019) Hepatic growth hormone - JAK2 - STAT5 signalling: Metabolic function, non-alcoholic fatty liver disease and hepatocellular carcinoma progression. *Cytokine* **124:** 154569.

111. Lin J-X, Leonard WJ. (2000) The role of Stat5a and Stat5b in signaling by IL-2 family cytokines. *Oncogene* **19:** 2566-2576.

112. Yoshii A, Constantine‐Paton M. (2010) Postsynaptic BDNF‐TrkB signaling in synapse maturation, plasticity, and disease. *Developmental neurobiology* **70:** 304-322.

113. Spoerri PE*, et al.* (1993) Neurotrophin-3 upregulates NGF receptors in a central nervous system glial cell line. *Neuroreport* **4:** 33-36.

114. Bathina S, Das UN. (2015) Brain-derived neurotrophic factor and its clinical implications. *Arch Med Sci* **11:** 1164-1178.

115. Miranda M, Morici JF, Zanoni MB, Bekinschtein P. (2019) Brain-Derived Neurotrophic Factor: A Key Molecule for Memory in the Healthy and the Pathological Brain. *Frontiers in Cellular Neuroscience* **13**.

116. Martinowich K, Manji H, Lu B. (2007) New insights into BDNF function in depression and anxiety. *Nature Neuroscience* **10:** 1089-1093.

117. Leidi M, Mariotti M, Maier JAM. (2010) The effects of silencing EDF-1 in human endothelial cells. *Atherosclerosis* **211:** 55-60.

118. Leidi M, Mariotti M, Maier JAM. (2010) EDF-1 contributes to the regulation of nitric oxide release in VEGF-treated human endothelial cells. *European Journal of Cell Biology* **89:** 654-660.

119. Bernardini D, Ballabio E, Mariotti M, Maier JAM. (2005) Differential expression of EDF-1 and endothelial nitric oxide synthase by proliferating, quiescent and senescent microvascular endothelial cells. *Biochimica et Biophysica Acta (BBA) - Molecular Cell Research* **1745:** 265-272.

120. Alvarez BV*, et al.* (2007) Identification and characterization of a novel mutation in the carbonic anhydrase IV gene that causes retinitis pigmentosa. *Invest Ophthalmol Vis Sci* **48:** 3459-3468.

121. Scozzafava A, Supuran CT. (2014) Glaucoma and the applications of carbonic anhydrase inhibitors. *Subcell Biochem* **75:** 349-359.

122. Garred P, Honoré C, Ma YJ, Munthe-Fog L, Hummelshøj T. (2009) MBL2, FCN1, FCN2 and FCN3—The genes behind the initiation of the lectin pathway of complement. *Molecular Immunology* **46:** 2737-2744.

123. Munthe-Fog L*, et al.* (2012) Variation in FCN1 affects biosynthesis of ficolin-1 and is associated with outcome of systemic inflammation. *Genes & Immunity* **13:** 515-522.

124. Chen X*, et al.* (2023) Identification of FCN1 as a novel macrophage infiltration-associated biomarker for diagnosis of pediatric inflammatory bowel diseases. *Journal of Translational Medicine* **21:** 203.

125. Katayama M*, et al.* (2018) Ficolin-1 is a promising therapeutic target for autoimmune diseases. *International Immunology* **31:** 23-32.

126. Pinte S*, et al.* (2016) Endothelial Cell Activation Is Regulated by Epidermal Growth Factor-like Domain 7 (Egfl7) during Inflammation *. *Journal of Biological Chemistry* **291:** 24017-24028.

127. Campagnolo L*, et al.* (2005) EGFL7 Is a Chemoattractant for Endothelial Cells and Is Up-Regulated in Angiogenesis and Arterial Injury. *The American Journal of Pathology* **167:** 275-284.

128. Nikolic I*, et al.* (2013) EGFL7 ligates αvβ3 integrin to enhance vessel formation. *Blood* **121:** 3041-3050.

129. Soncin F*, et al.* (2003) VE-statin, an endothelial repressor of smooth muscle cell migration. *Embo j* **22:** 5700-5711.

130. Ricagno S, Caccia S, Sorrentino G, Antonini G, Bolognesi M. (2009) Human neuroserpin: structure and time-dependent inhibition. *J Mol Biol* **388:** 109-121.

131. Yepes M, Lawrence DA. (2004) Neuroserpin: a selective inhibitor of tissue-type plasminogen activator in the central nervous system. *Thromb Haemost* **91:** 457-464.

132. Yepes M, Lawrence DA. (2004) Tissue-type plasminogen activator and neuroserpin: a well-balanced act in the nervous system? *Trends Cardiovasc Med* **14:** 173-180.

133. Roberts HR, Hoffman M, Monroe DM. (2006) A Cell-Based Model of Thrombin Generation. *Semin Thromb Hemost* **32:** 032-038.

134. Boonstra J*, et al.* (1995) The epidermal growth factor. *Cell Biology International* **19:** 413-430.

135. He X*, et al.* (2021) LYSMD3: A mammalian pattern recognition receptor for chitin. *Cell Reports* **36:** 109392.

**FIGURE LEGENDS**

**Supplemental Figure 1. A Volcano plot demonstrating the log2 change in biomarkers between MIS-C and SCNS patients.** The top 58 biomarkers (P<0.0001, FDR adjusted P<0.005) are highlighted, with those coloured green demonstrating an increase in protein expression in MIS-C compared to SCNS patients, while those in red demonstrate a decrease in MIS-C protein expression relative to SCNS patients.

**Supplemental** **Figure 2. Identification of key significant blood proteins in MIS-C patients. A)** Subjects plotted in two dimensions, following t-SNE dimensionality reduction of all 2888 measured, showed cluster separation of MIS-C patients, SCNS patients, and healthy control subjects with one outlier. Classification accuracy represents a binary classifier comparing MIS-C and a combined group of SCNS and Healthy Controls. **B)** Subjects plotted in two dimensions, following t-SNE dimensionality reduction of the top 58 significant proteins (P<0.0001, FDR adjusted P<0.005), showed a separation cluster of MIS-C patients, SCNS patients, and healthy control subjects with one outlier. **C)** A heatmap demonstrated the pairwise cosine similarity between the subjects’ complete protein profiles. A greater cosine similarity measure between subjects indicated similar protein profiles, while a smaller measure indicated large differences between profiles (the measure was pseudocolored on the bar scale). The protein profile of healthy control subjects is more homogenous and distinct from other cohorts, with minimal distinction between MIS-C and SCNS patients. **D)** A heatmap demonstrated the pairwise cosine similarity between participants’ top 58 protein profiles. A greater cosine similarity measure between subjects indicated similar protein profiles, while a smaller measure indicated large differences between profiles (the measure was pseudocolored on the bar scale). The protein profile of MIS-C patients was distinctively different from other cohorts, with some homogeneity across the different days.

**Supplemental Figure 3. Significant Protein Expression changes in MIS-C Patients over 3 PICU Days.** The leading 58 proteins were compared with Wilcoxon Signed Rank Test across days 1, 2, and 3 of PICU stay. **A-C)** Boxplot depicting changes in protein expression for SERPINA1, PRDX6, and SOST. For all three proteins, expression decreased with PICU length of stay (P<0.05). There were no significant changes in expression between days 1 and 2 or days 2 and 3.

**Supplemental** **Figure 4. Significant Correlations Between Protein Expression in MIS-C Patients and Continuous Clinical Variables.** The 58 leading proteins of MIS-C on PICU Day 1 were compared to continuous clinical and demographic variables; the significant correlations are shown (P<0.05). Blue points are MIS-C patient measurements; the green-filled area represents the 25th percentile to 75th percentile protein expression range of healthy control subjects. The Spearman correlation Rho statistic (𝜌) and the significance value of the comparison are shown. **A-B)** Plots demonstrating a positive correlation between hospital length of stay and LTA4H and PTN, respectively; however, compared to healthy controls, LTA4H expression was greater in MIS-C patients on Day 1, and PTN expression was lower in MIS-C patients on Day 1 **C-D)** Plots demonstrating a negative correlation between PICU length of stay relative to ANGPT1 and PPBP; for both proteins, lower expression was associated with a greater PICU length of stay. **E-G)** Plots of BDNF, EGF, and FCN1 expression demonstrate a negative correlation with PIM2 mortality risk, such that greater expression was associated with lower PIM2 mortality risk. **H)** Plot of HLA-DRA protein expression demonstrating a positive correlation with the highest recorded sPELOD score, such that increased expression was associated with a greater sPELOD score.

**Supplemental** **Figure 5. Protein Expression in MIS-C was Associated with Significantly Different Categorical Clinical Variables.** The 58 leading proteins of MIS-C on PICU Day 1 were compared to measured categorical clinical and demographic variables; the significant correlations are shown (P<0.05). The green filled area represents the 25th percentile to 75th percentile protein expression range of healthy control subjects. **A-C)** Boxplots demonstrating the difference in HLA-DRA, PTN, and EGF protein expression in those that received Inotrope/Vasopressor intervention. HLA-DRA and PTN expression were elevated in those that received the intervention, while EGF expression decreased expression. **D)** Boxplot demonstrating elevated STAT5B expression in obese inpatients compared to non-obese inpatients.

**Supplemental** **Figure** **6.** **Frequency of protein expression in major organ systems and cell types.** **A)** A bar plot demonstrating the percentage of proteins that were expressed in specific organ systems as determined by Natural Language Processing. There were 29 proteins (50%) that had UniProt organ system expression information. The organ system classification included tissue, multi-level tissue, and anatomical system entities. **B)** A bar plot demonstrating the percentage of proteins that were expressed in specific cell types as determined by Natural Language Processing. There were 19 proteins (33%) that had UniProt cell type expression information. Only those cell types with percentages greater than 5% are shown for clarity of visualization.

**Supplemental Figure 1:
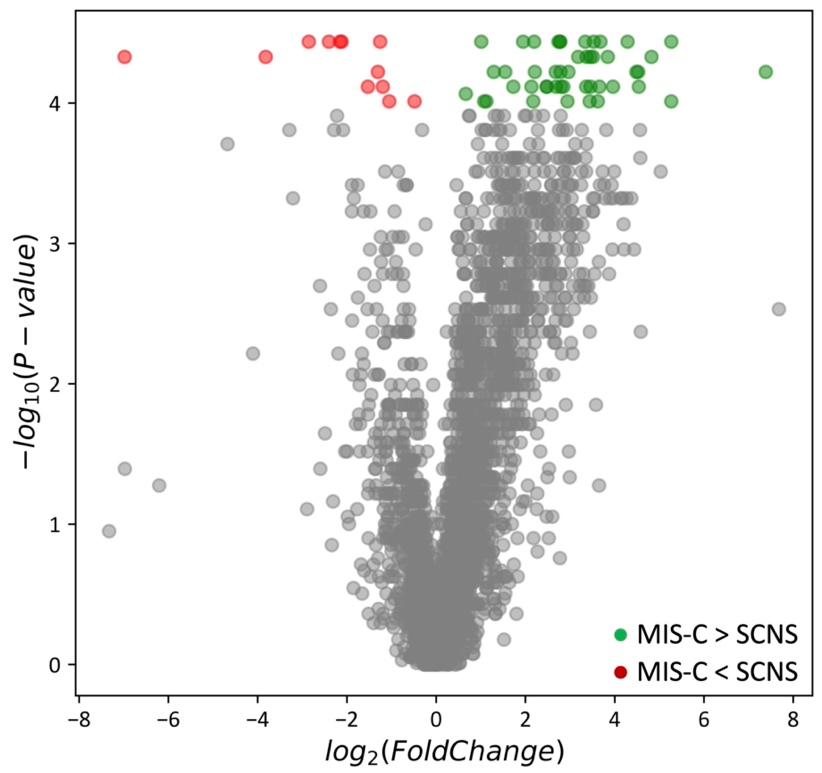
**

**S****upplemental Figure 2:**


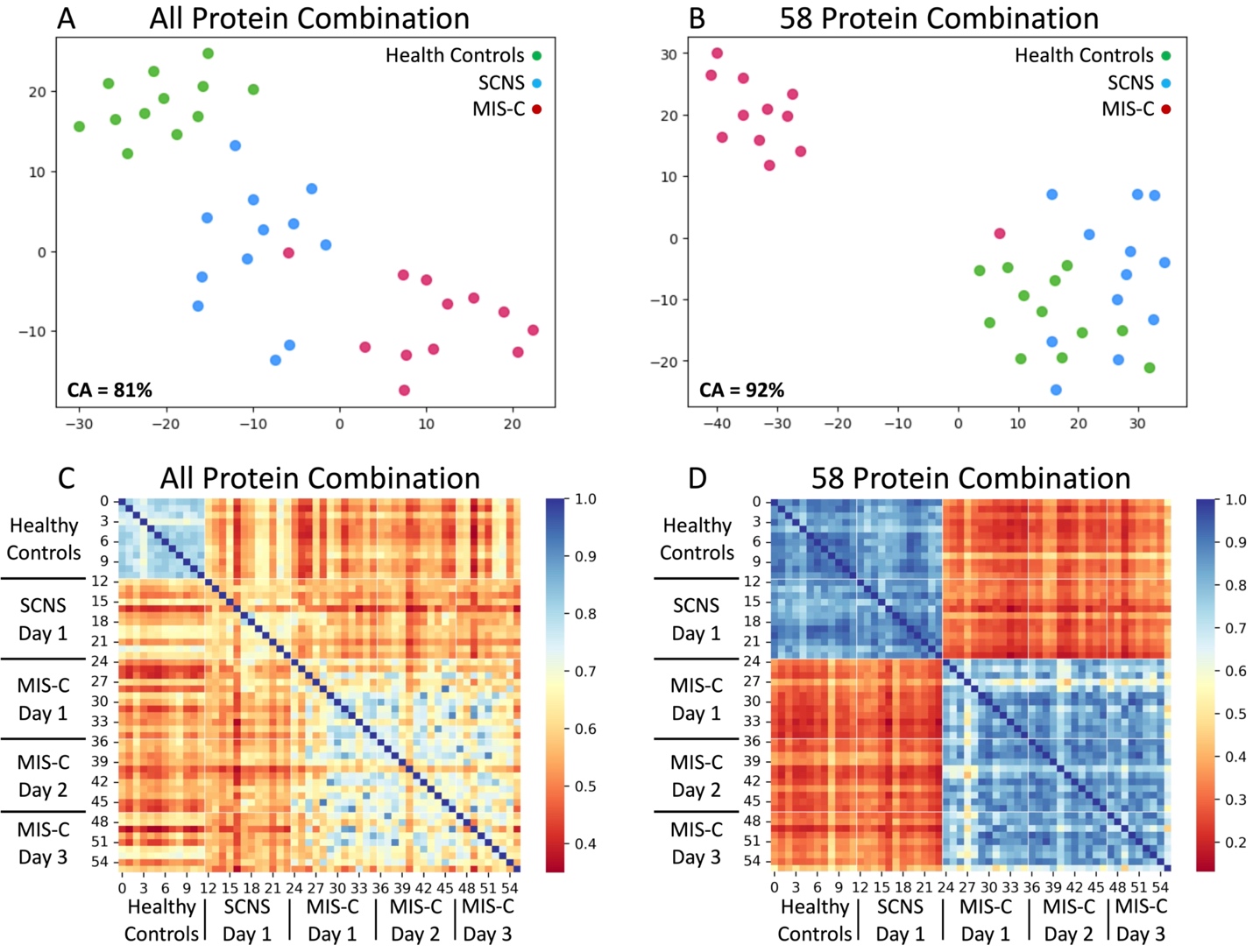


**Supplemental Figure 3:**

**
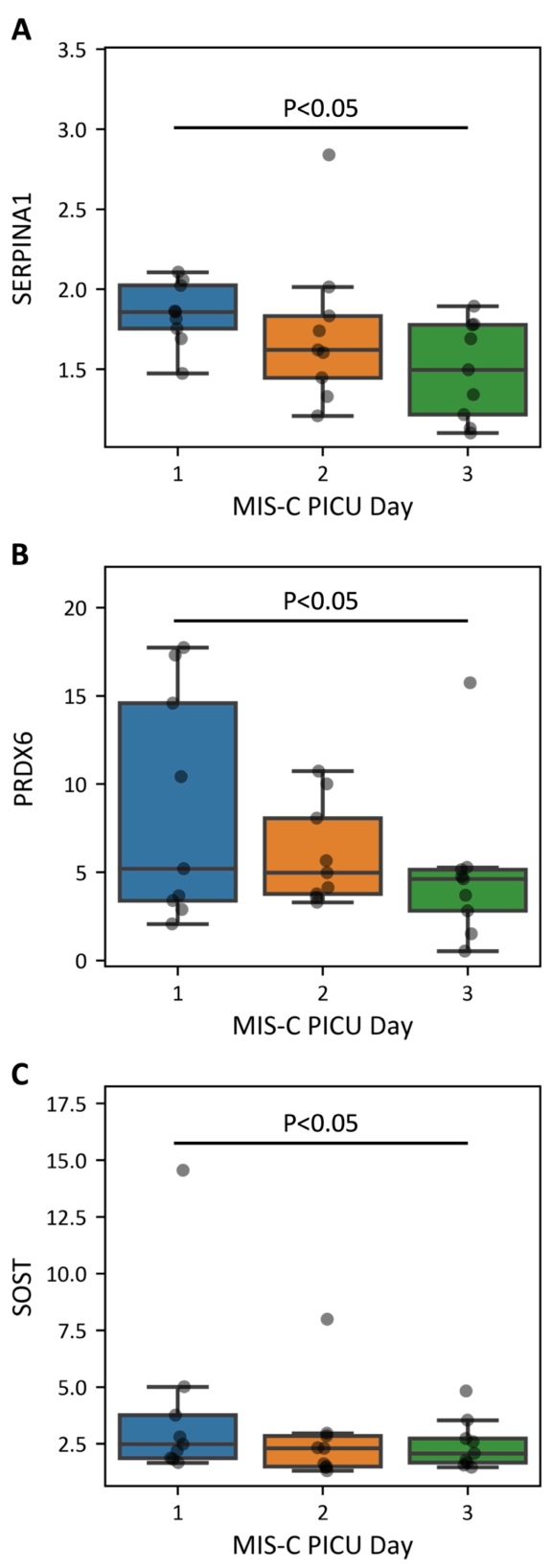
**

**Supplemental Figure 4:**

**
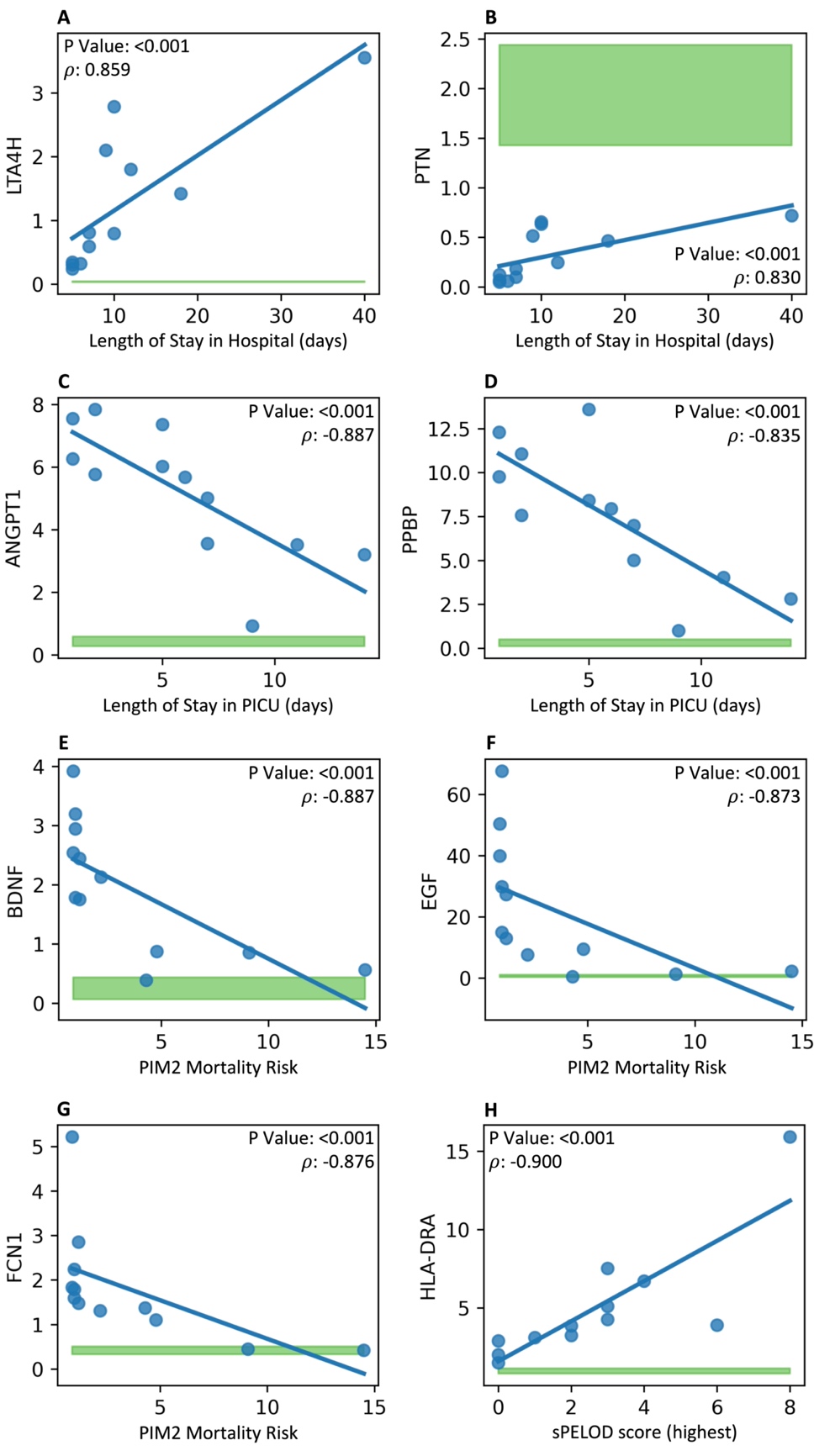
**

**Supplemental Figure 5:** **
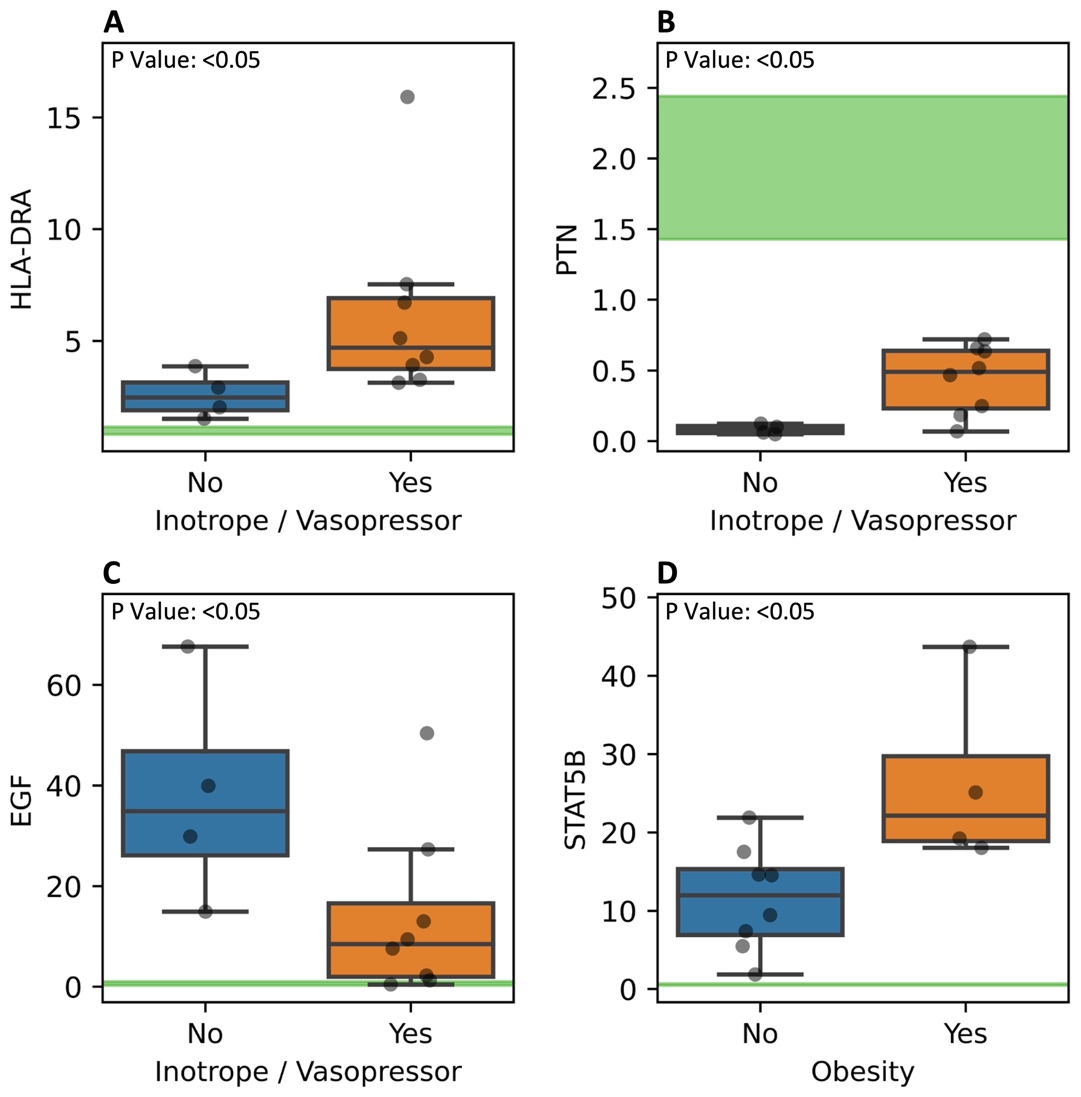
**

**
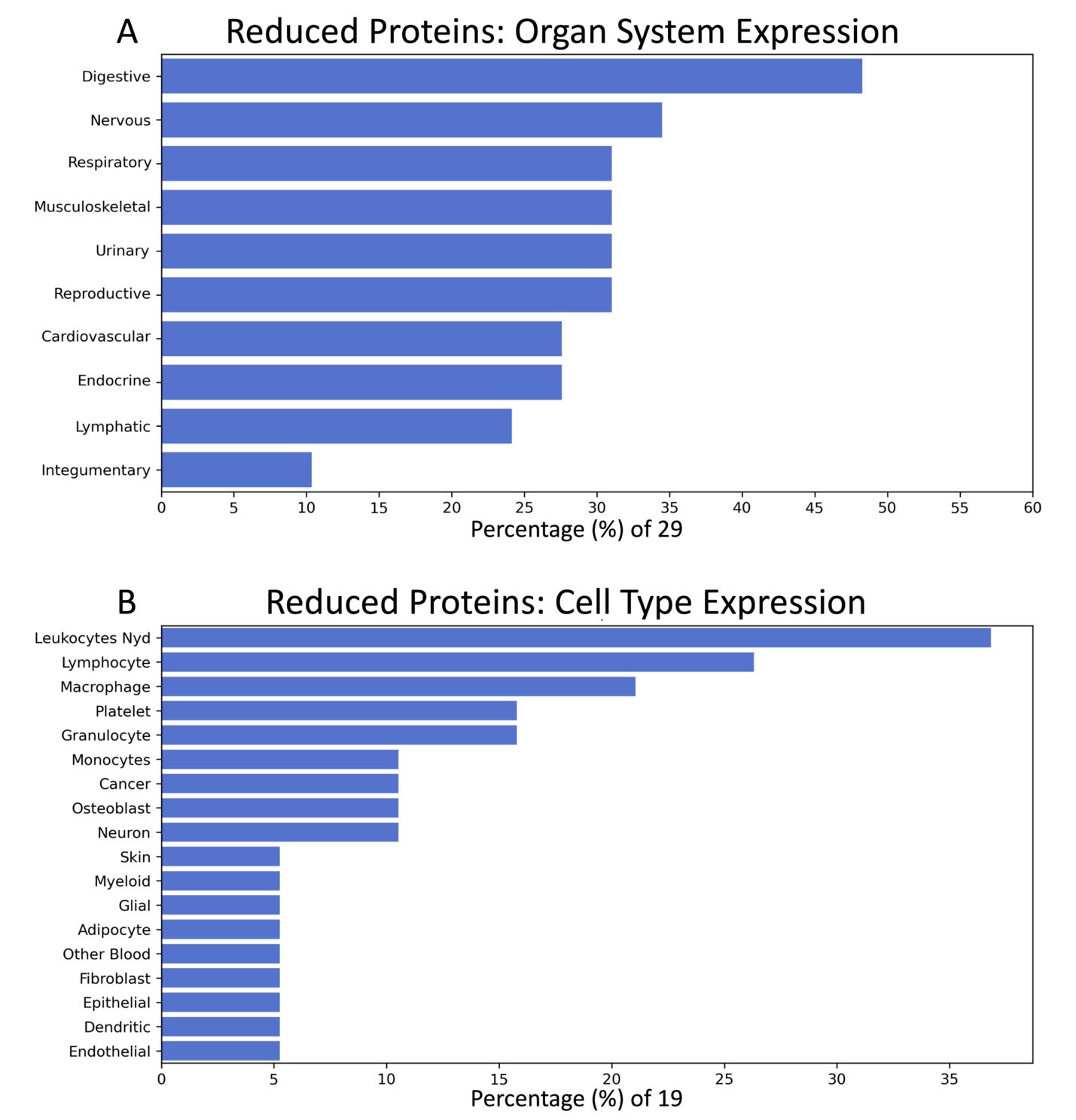
Supplemental Figure 6:**
